# Excessive fat expenditure in MCT-induced heart failure rats is associated with BMAL1/REV-ERBα circadian rhythmic loop disruption

**DOI:** 10.1038/s41598-024-58577-8

**Published:** 2024-04-07

**Authors:** Dufang Ma, Yiwei Qu, Tao Wu, Xue Liu, Lu Cai, Yong Wang

**Affiliations:** 1 First Clinical Medical College, Shandong University of Traditional Chinese Medicine, Jinan, 250014 Shandong China; 2 Department of Cardiology, Shandong University of Traditional Chinese Medicine Affiliated Hospital, No. 16369 Jingshi Road, Lixia District, Jinan, 250014 Shandong China

**Keywords:** Heart failure, Cardiac cachexia, Fat expenditure, Circadian clock, BMAL1/REV-ERBα circadian rhythmic loop, White adipose lipolysis, Beiging of white adipose tissue, Brown adipose thermogenesis, Biochemistry, Physiology, Cardiology, Diseases

## Abstract

Fat loss predicts adverse outcomes in advanced heart failure (HF). Disrupted circadian clocks are a primary cause of lipid metabolic issues, but it's unclear if this disruption affects fat expenditure in HF. To address this issue, we investigated the effects of disruption of the BMAL1/REV-ERBα circadian rhythmic loop on adipose tissue metabolism in HF.50 Wistar rats were initially divided into control (n = 10) and model (n = 40) groups. The model rats were induced with HF via monocrotaline (MCT) injections, while the control group received equivalent solvent injections. After establishing the HF model, the model group was further subdivided into four groups: normal rhythm (LD), inverted rhythm (DL), lentivirus vector carrying Bmal1 short hairpin RNA (LV-Bmal1 shRNA), and empty lentivirus vector control (LV-Control shRNA) groups, each with 10 rats. The DL subgroup was exposed to a reversed light–dark cycle of 8 h: 16 h (dark: light), while the rest adhered to normal light–dark conditions (light: dark 12 h: 12 h). Histological analyses were conducted using H&E, Oil Red O, and Picrosirius red stains to examine adipose and liver tissues. Immunohistochemical staining, RT-qPCR, and Western blotting were performed to detect markers of lipolysis, lipogenesis, and beiging of white adipose tissue (WAT), while thermogenesis indicators were detected in brown adipose tissue (BAT). The LD group rats exhibited decreased levels of BMAL1 protein, increased levels of REV-ERBα protein, and disrupted circadian circuits in adipose tissue compared to controls. Additionally, HF rats showed reduced adipose mass and increased ectopic lipid deposition, along with smaller adipocytes containing lower lipid content and fibrotic adipose tissue. In the LD group WAT, expression of ATGL, HSL, PKA, and p-PKA proteins increased, alongside elevated mRNA levels of lipase genes (Hsl, Atgl, Peripilin) and FFA β-oxidation genes (Cpt1, acyl-CoA). Conversely, lipogenic gene expression (Scd1, Fas, Mgat, Dgat2) decreased, while beige adipocyte markers (Cd137, Tbx-1, Ucp-1, Zic-1) and UCP-1 protein expression increased. In BAT, HF rats exhibited elevated levels of PKA, p-PKA, and UCP-1 proteins, along with increased expression of thermogenic genes (Ucp-1, Pparγ, Pgc-1α) and lipid transportation genes (Cd36, Fatp-1, Cpt-1). Plasma NT-proBNP levels were higher in LD rats, accompanied by elevated NE and IL-6 levels in adipose tissue. Remarkably, morphologically, the adipocytes in the DL and LV-Bmal1 shRNA groups showed reduced size and lower lipid content, while lipid deposition in the liver was more pronounced in these groups compared to the LD group. At the gene/protein level, the BMAL1/REV-ERBα circadian loop exhibited severe disruption in LV-Bmal1 shRNA rats compared to LD rats. Additionally, there was increased expression of lipase genes, FFA β oxidation genes, and beige adipocyte markers in WAT, as well as higher expression of thermogenic genes and lipid transportation genes in BAT. Furthermore, plasma NT-proBNP levels and adipose tissue levels of NE and IL-6 were elevated in LV-Bmal1 shRNA rats compared with LD rats. The present study demonstrates that disruption of the BMAL1/REV-ERBα circadian rhythmic loop is associated with fat expenditure in HF. This result suggests that restoring circadian rhythms in adipose tissue may help counteract disorders of adipose metabolism and reduce fat loss in HF.

## Introduction

The course and prognosis of chronic heart failure (HF) are closely associated with an individual's nutritional status. Although obesity has been regarded as a traditional risk factor for developing HF, it has been reported that overweight or class 1 obese individuals have better survival than patients with normal weight^1^. This curious phenomenon was termed the “obesity paradox”. In comparison, cachexia, which is a well-known risk factor for death in patients with end-stage chronic HF^2^ is accompanied by significant muscle loss, fat wasting and bone tissue loss^3^. Melenovsky et al.^4^ reported that fat wasting was more prevalent among patients with right ventricular dysfunction than among those with left ventricular dysfunction. Several clinical studies have shown that adipose tissue is the main source of energy expenditure in malignant disease, rather than muscle; furthermore, fat loss and atrophy predict adverse outcomes in advanced HF^4,5^. Indeed, fat wasting increases susceptibility to infection and aggravates ectopic fat deposition caused by excessive lipolysis^6^. Study suggested that enhanced lipolysis and fat oxidation, decreased lipogenesis, impaired lipid storage, and increased beiging/brown adipocyte thermogenesis may be underlying causes of excessive fat expenditure^7^. Additionally, studies have suggested that excessive sympathetic activity and increased proinflammatory cytokines are the main factors contributing to fat expenditure in HF^3^.

The circadian clock developed into an independent system of timekeeping that supports bodily processes by synchronizing the necessary metabolic program and harmonizing behavioral patterns with the progression of day and night. At the molecular level, control of circadian rhythmicity is thought to involve the interaction of two major autoregulatory transcription-translation feedback loops. The central feedback loop includes circadian locomotor output cycles kaput (CLOCK) and brain and muscle aryl hydrocarbon receptor nuclear translocator-like protein 1 (BMAL1). CLOCK and BMAL1 interact to form a heterodimer that drives the expression of Period (PER) and Cryptochrome (CRY). In turn, the PER and CRY proteins, which accumulate in the cytoplasm, then translocate to the nucleus, thus feedback inhibiting the activity of the CLOCK:BMAL1 heterodimer^8^. Additionally, the CLOCK: BMAL1 heterodimer promotes the expression of nuclear receptor reverse ERB (REV-ERB), which negatively regulates BMAL1 expression^9^. Molecular clocks are found in adipose tissue and are known to regulate lipid metabolism in adipocytes^10^.

An increasing body of evidence indicates that insulin resistance and metabolic inflammation brought on by a disrupted circadian clock increase the risk of obesity and type 2 diabetes^11^. It has been demonstrated that persistent jet lag in environments with variable lighting induces obesity in both shift workers and mice^12,13^. Additionally, it has been proposed that the expression of lipogenic and adipogenesis genes in white adipose tissue (WAT) decreases and loses rhythmicity in mice with cancer cachexia caused by C26 tumours, suggesting a decrease in lipid accumulation. Furthermore, at two time intervals, there was an increase in the protein levels of the lipolytic enzymes perilipin and adipose triglyceride lipase (ATGL), indicating greater lipolysis^14^. These studies have shown that fat accumulation in obesity and fat expenditure in cancer cachexia are associated with circadian rhythm disruption. Similarly, in HF, suppressing excessive fat expenditure is an important strategy for improving HF prognosis and preventing cachexia, but how circadian rhythm factors affect fat metabolism in HF has not been investigated.

BMAL1 is the 'commander' of the biological clock, responsible for initiating transcription-translation feedback loops, and is a core circadian transcription factor^15–17^. Deficiency of BMAL1 in mice leads to a complete loss of behavioural and physiological circadian rhythmicity^18^. Therefore, Bmal1-deficient mice have been widely used for circadian rhythm studies. Deletion of Bmal1 in mice resulted in decreased capability of novo lipogenesis and fat storage^19^ and increased levels of circulating FFAs and triglycerides (TGs) due to excessive lipolysis^20^. BMAL1, together with its negative regulator REV-ERBα, constitutes the central feedback loop of the biological clock, causing circadian changes in life activities. Administration of the REV-ERBα agonist SR9009 inhibits the expression of genes involved in lipid synthesis and storage in WAT, reducing fat accumulation^21^. However, there is currently insufficient data to indicate a connection between the BMAL1/REV-ERBα circadian rhythmic loop and fat expenditure in HF. To address this issue, we generated HF rats by monocrotaline (MCT)-induced pulmonary hypertension and investigated the relationship between the BMAL1/REV-ERBα circadian rhythmic loop disruption and fat expenditure in MCT-induced HF.

## Materials and methods

### Animals and reagents

A total of 50 specific pathogen-free (SPF) adult male Wistar rats weighing 260–280 g were purchased from Beijing Vital River Corporation (Permit No. SCXK (JING) 2021-0011). All rats were housed in separate cages under a 12 h light–dark cycle at 23 ± 1 °C and 50% relative humidity, and food and water were available ad libitum. The animal study was approved by the Experimental Animal Ethics Committee of Shandong University of Traditional Chinese Medicine (Licence No. 2021-30). All methods were performed according to ARRIVE guidelines. This study complied with the Regulations on the Management of Laboratory Animals issued by the State Science and Technology Commission and the Implementing Rules for the Management of Medical Laboratory Animals issued by the Health Planning Commission, as well as the Statutes of the Experimental Animal Ethics Committee of the Affiliated Hospital of Shandong University of Traditional Chinese Medicine, and the conditions of the personnel and equipment were in accordance with the requirements.

After 1 week of adaptive feeding, 40 rats were intraperitoneally injected with monocrotaline (60 mg/kg, Lot No. MUST-21111217, Chengdu Must Bio-Technology Co., Ltd) to induce HF. Meanwhile, rats in the control group were intraperitoneally injected with 3 ml/kg solvent (saline: alcohol = 8:2). MCT-induced pulmonary hypertension is an experimental model of cardiac cachexia since it leads to progressive HF and cachexia very rapidly^22,23^. After 4 weeks of MCT injection, we measured body weight and cardiac function to demonstrate successful HF modelling. Subsequently, the rats with HF were randomly assigned to four groups: the normal rhythm group (LD group) (n = 10), inverted rhythm group (DL group) (n = 10), empty lentivirus vector control group (LV-Control shRNA group) (n = 10) and lentivirus vector carrying Bmal1 short hairpin RNA group (LV-Bmal1 shRNA group) (n = 10). Rats without MCT treatment were assigned to the control group (n = 10). Rats in the DL group were housed in a dark: light 8 h:16 h cycle to disrupt normal dark–light cycle, with lights off at 7 A.M and lights on at 3 P.M. Rats in the other four groups were housed in a light: dark 12 h:12 h cycle, with lights on at 7 A.M. and light off at 7 P.M^24^. Meanwhile, rats in the LV-Bmal1 shRNA group received a focal point injection of lentivirus carrying Bmal1 shRNA in inguinal fat and epididymal fat to inhibit the expression of the Bmal1, and 1.5 × 10^7^ TU was injected at each point. Rats in the LV-Control shRNA group were injected with empty lentiviral vehicle. Rats in the control group and DL group, LV-Bmal1 shRNA group and LV-Control shRNA group were fed in pairs to match the daily food intake of the LD group^25^.

### Construction of the lentivirus LV-Bmal1 shRNA

Lentivirus-expressing short hairpin RNA (shRNA) targeting the sequence of the Bmal1 gene (5’-ATCCTCAATTATAGCCAGAAT-3’) and a negative control (5’-TTCTCCG AACGTGTCACGT-3’) were constructed and synthesized by GenePharma Biotech (Shanghai, China). Correct insertion of shRNA cassettes was confirmed by direct DNA sequencing. The titer of LV-Bmal1 shRNA was 2E + 9 TU/mL.

### Ultrasound cardiography

After the rats were anaesthetized at the 4th week after MCT injection, indicators reflecting heart function, including left ventricular ejection fraction (EF) and left ventricular fractional shortening (FS) as well as right ventricular fractional area change (RV-FAC), were recorded. RV-FAC was calculated by RV-FAC = (end-diastolic area (EDA)-end-systolic area (ESA))/EDA × 100%.

### Tissue collection

At the end of the 8th week after injection of MCT, each rat was anaesthetized with pentobarbital sodium (40 mg/kg, i.p.) at 7:00 A.M. Blood samples collected from the inferior vena cava were centrifuged at 3000 rpm for 15 min at 4 °C to separate the plasma and preserved at − 80 °C for ELISA. WAT, including epididymal fat and inguinal fat, and brown adipose tissue (BAT) in the scapula were removed as quickly as possible on ice and recorded as the total fat weight. The liver tissue, epididymal fat and brown fat in the scapula were immediately stored in liquid nitrogen for subsequent experimental testing^25^.

### Histological analysis

Epididymal fat and brown fat were fixed in 4% paraformaldehyde for 12 h, embedded in paraffin, cut into 4-µm thick sections and subsequently stained with HE staining and Oil Red O staining (SERVICEBIO, Wuhan, China). Additionally, epididymal fat was stained with picrosirius red (PSR). Finally, tissue sections were viewed under a NIKON microscope (NIKON Eclipse E100, NIKON, Tokyo, Japan). Images were obtained from three random regions of each slice. The analysis was performed with a NIKON DS-U3 (NIKON, Tokyo, Japan).

### Immunohistochemical staining

Paraffin-embedded sections were cut into 4 μm thick sections, deparaffinized with xylene and incubated with hydrogen peroxide to block endogenous peroxidase. 3% BSA was added dropwise to the histochemical circle to cover the tissue evenly, and closed at room temperature for 30 min. Then, the sections were incubated with anti-uncoupling protein-1 (UCP-1) (1:200, Cat No. GB112174, SERVICEBIO, Wuhan, China) primary antibodies overnight at 4 ℃ and subsequently incubated with goat anti-rabbit secondary antibody (1:200, Cat No. G1213, SERVICEBIO, Wuhan, China). Sections were first developed with freshly prepared DAB colour development solution and then counterstained with haematoxylin before being mounted. The stained slides were observed under a NIKON microscope (NIKON Eclipse E100, NIKON, Tokyo, Japan). Images collected from three random fields per slice were finally analysed using a NIKON DS-U3 (NIKON, Tokyo, Japan)^25^.

### Enzyme-linked immunosorbent assay (ELISA)

The levels of TGs, FFAs and NT-proBNP in blood and interleukin-6 (IL-6) and norepinephrine (NE) in both WAT and BAT were measured using high-sensitivity ELISA kits (TG (Cat No. BC0625, SOLARBIO, Beijing, China), FFA (Cat No. BC0595, SOLARBIO, Beijing, China), NT-proBNP (Cat No. CSB-E07972r, CUSABIO, Wuhan, China), IL-6 (Cat No. JYM0646Ra, JYM, Wuhan, China), NE (Cat No. JYM0587Ra, JYM, Wuhan, China)). All testing steps were performed according to the product instructions attached to the product.

### Real-time quantitative PCR (RT‒qPCR)

Total RNA was extracted from the frozen epididymal fat and brown fat in the scapula by the TRIzol method using SparkZol Reagent (Cat No. AC0101, SPARKJADE, Jinan, China) and reverse transcribed by using the SPARKscript II RT Plus Kit (Cat No. AG0304, SPARKJADE, Jinan, China). Gene expression was analysed by quantitative PCR performed using Light Cycler 480 SYBR Premix Ex Taq II (Roche, Basel, Switzerland).

The mRNA expression levels of hormone sensitive lipase (Hsl), Atgl, Peripilin, carnitine palmitoyl transferase 1 (Cpt1), acyl-CoA, stearoyl-CoA desaturase 1 (Scd1), fatty acid synthase (Fas), monoacylglycerol acyltransferase (Mgat), diacylglycerol acyltransferase-2 (Dgat2), Cd137, Tbx-1, Zinc finger protein 1 (Zic-1), Ucp-1, CD36, Fatty Acid Transport Protein-1 (Fatp-1), Peroxisome Proliferator-Activated Receptor Gamma (Pparγ), proliferator-activated receptor-γ coactivator 1α (Pgc-1α), Clock, Bmal1, and Rev-erbα were detected, and β-actin was used for normalization. The relative gene expression was analysed using the comparative Ct method formula, 2^-ΔΔCT^^26^. The sequences of the forwards/reverse primers (synthesized by SPARKJADE China) are listed in Table 1.

**Table 1:** The sequences of the forwards/reverse primers list.

Genes	Primer sequences
β-actin	Forward 5’-CCCATCTATGAGGGTTACGC’
	Reverse 5’-TTTAATGTCACGCACGATTTC’
Hsl	Forward 5’-GTCCCTCATCGCCCTCAAAGAAG’
	Reverse 5’-GCCAGCCACAACCTAGCAGAAC’
Atgl	Forward 5’-TGGATGAAGGAGCAGACAGGTAGC’
	Reverse 5’-AGTGGCACAGACGGCAGAGAC’
Periphilin	Forward 5’-ACCCTCAAGTTCGTCAGCAGTTTC’
	Reverse 5’-TCTCAGCCTCAGCGAGTTCCTTC’
Cpt1	Forward 5’-CAGGAGAGTGCCAGGAGGTCATAG’
	Reverse 5’-TGCCGAAAGAGTCAAATGGGAAGG’
Scd1	Forward 5’-TGTCAAAGAGAAGGGCGGAAAGC’
	Reverse 5’-CAGGATGAAGCACATGAGCAGGAG’
Fas	Forward 5’-GTCCTGCCTCTGGTGCTTGC’
	Reverse 5’-TTCACGAACGCTCCTCTTCAACTC’
Dgat2	Forward 5’-CCGCAGCGAGAACAAGAATA’
	Reverse 5’-GACCCACTGTGAGACTGAGATGAC’
Mgat	Forward 5’-GCAGCCAGACCACCGTAAGTTC’
	Reverse 5’-TCCAGATCATCCTCCACTACCACAG’
Cd137	Forward 5’-AAGCCTTGCTCCTCTACCCA’
	Reverse 5’-CGTTCCCAAGCCACAGTTT’
Tbx-1	Forward 5’-CGGTGAAGAAGAACCCAAAG’
	Reverse 5’-TCCACAGGCACAAAGTCCA’
Zic1	Forward 5’-GTCCTCTTCTCAGGGCTCACAG’
	Reverse 5’-GCTGGTGGTCGGGTTGTCT’
Ucp-1	Forward 5’-GGCATCCAGAGGCAAATCA’
	Reverse 5’-GTCATCAAGCCAGCCGAGA’
Clock	Forward 5’-ACGGCGAGAACTTGGCATTGAG’
	Reverse 5’-TGGAGGAGGCAGAAGGAGTTGG’
Bmal1	Forward 5’-AGGACTTCGCCTCCACCTGTTC’
	Reverse 5’-GCCCTCATTGTCTGGTTCACTGTC’
Rev-erbα	Forward 5’-CGGTCTACGGCAAGGCAACAC’
	Reverse 5’-CAAATTCTACCACCTCCCGCACAG’
Acyl-CoA	Forward 5’-AAATCAAGCAAAGCGAACCAGAACC’
	Reverse 5’-CGAAGTGGAAGGCATAGGCAGTG’
Cd36	Forward 5’-TTCTTCCAGACGCCTTTGC’
	Reverse 5’-CTTCTTTGCACTTGCCAATGTCCAG’
Fatp-1	Forward 5’-GGCTGTGTATGGAGTGGCTGTG’
	Reverse 5’-AGGAGGATGGGCTGGGCATAGG’
PParγ	Forward 5’-TCTGTGGACCTCTCTGYGATGGATG’
	Reverse 5’-GTCAGCTCTTGTGAACGGGATGTC’
Pgc-1α	Forward 5’-ACCGCACACATCGCAATTCTCC’
	Reverse 5’-AGACTCCCGCTTCTCATACTCTCTG’

## Western blot analysis

Protein was collected from the epididymal fat and brown fat in the scapula using the Minute Animal Adipose Tissue Protein Extraction Kit (INVENT, Minnesota, USA)^25^. Additionally, an enhanced bicinchoninic acid (BCA) protein assay kit (CWBIO, Jiangsu, China) was used to measure the concentrations of protein. And the protein concentration was prepared into a normalised protein system with a concentration of 2 µg/µL by adding PBS and loading buffer. Subsequently, 10% sodium dodecyl sulfate–polyacrylamide gel electrophoresis (SDS-PAGE) was used to extract 20 µg of protein from each group, and the protein was then transferred onto PVDF membranes (MILLIPORE, Massachusetts, USA) for blotting. The membranes were then incubated with 5% skimmed milk in TBST for 1 h at room temperature. Following that, the immunoblots were washed and then probed with primary antibodies overnight for 8 h at 4 °C against glyceraldehyde 3-phosphate dehydrogenase (β-ACTIN) (1:1000, Cat No. 20536-1-AP, PROTEINTECH, Wuhan, China), protein kinase A (PKA) (1:1000, Cat No. ab32390, ABCAM, Cambridge, United Kingdom), phospho-PKA (p-PKA) (1:500, Cat No. Bs-3725r, BIOSS, Beijing, China), HSL (1:1000, BIOSS, Cat No. Bs-0455r), ATGL (1:2000, ABCAM, Cat No. ab109251), REV-ERBα (1:1000, SANTA CRUZ, Cat No. sc-393215) and BMAL1 (1:1000, ABCAM, Cat No. ab235577). Then, the membranes were washed and incubated with the secondary antibody of the corresponding species. Then, the protein bands were visualized with a Sensitivity ECL Chemiluminescence Detection Kit (PROTEINTECH, Wuhan, China). Finally, the protein content was analysed by densitometric quantification using Tanon 5200 Fully Automated Chemiluminescent Image Analysis System (TANON, Shanghai, China). Supplementary file contains the original images of all gels.

### Statistical analysis

The obtained data were statistically analysed by GraphPad Prism 8.0. Data are presented as the mean and standard deviation (SD). Differences were analysed using the single-factor analysis of variance method (ANOVA) or nonparametric Kruskal–Wallis test according to the variances. P < 0.05 was defined as statistically significant.

### Ethical approval and consent to participate

The animal study was approved by the Experimental Animal Ethics Committee of Shandong University of Traditional Chinese Medicine (Licence No. 2021-30).

## Results

### MCT injection induced progressive ventricular dysfunction

**Figure 1:**
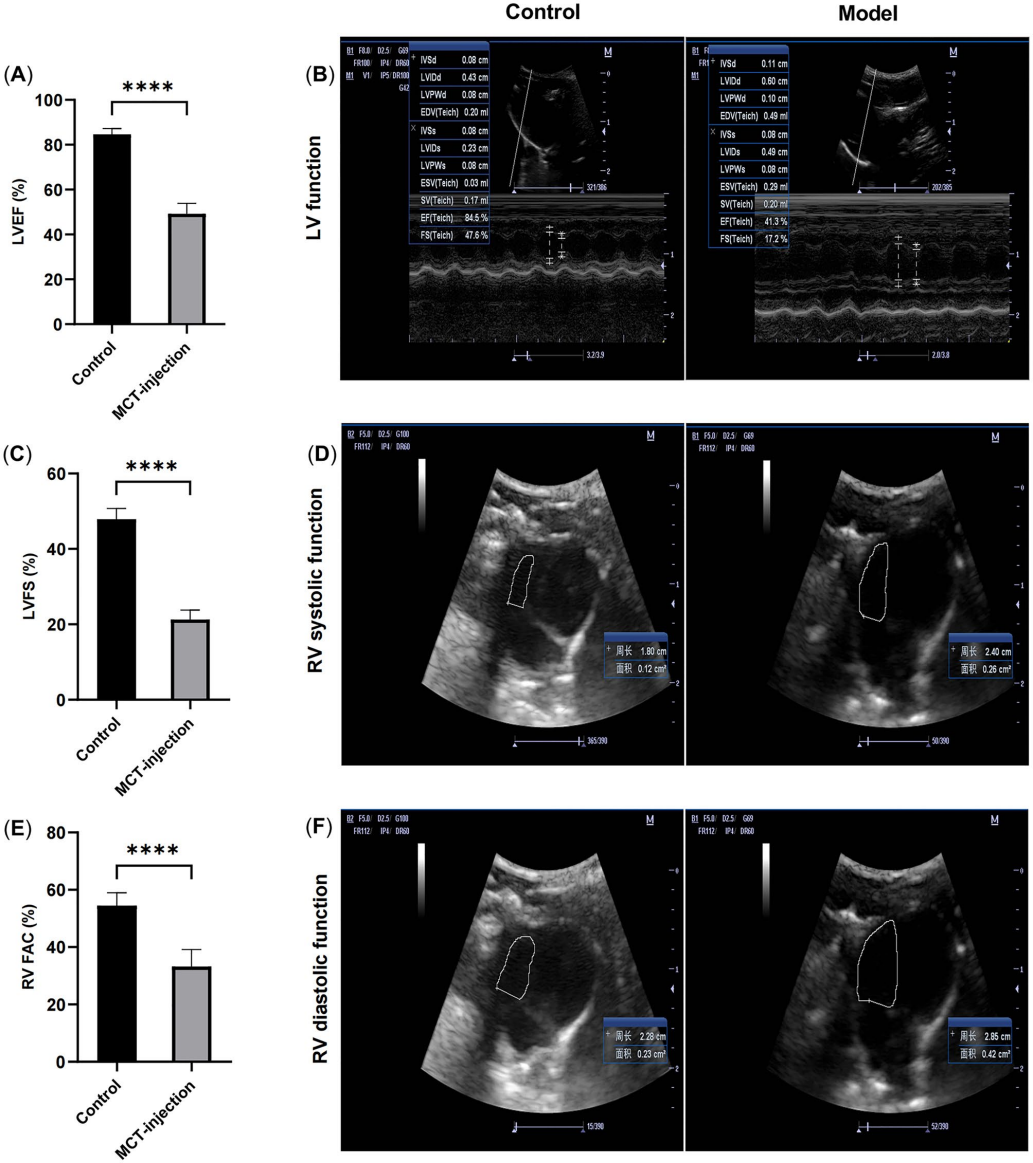
MCT injection induced decreased right and left ventricular function. (**A**) Left ventricular ejection fraction (EF). (**B**) Representative images of echocardiography reflecting left ventricular function. (**C**) Left ventricular fractional shortening (FS). (**D**,**F**) Representative images of echocardiography reflecting right ventricular function. (**E**) Right ventricular fractional area change. Data are mean ± SD, n = 6. ****P < 0.0001.

At the end of the 4th week after MCT injection, as shown in Fig. 1, compared with the control group, the model group showed a significant decrease in left and right ventricular function, as demonstrated by reduced indicators, including LVEF, LVFS and RV-FAC (Fig. 1A,C,E, P < 0.01). This indicates that rats treated with MCT exhibited significant HF.

### BMAL1/REV-ERBα circadian rhythmic loop disruption was observed in adipose tissue of HF

**Figure 2:**
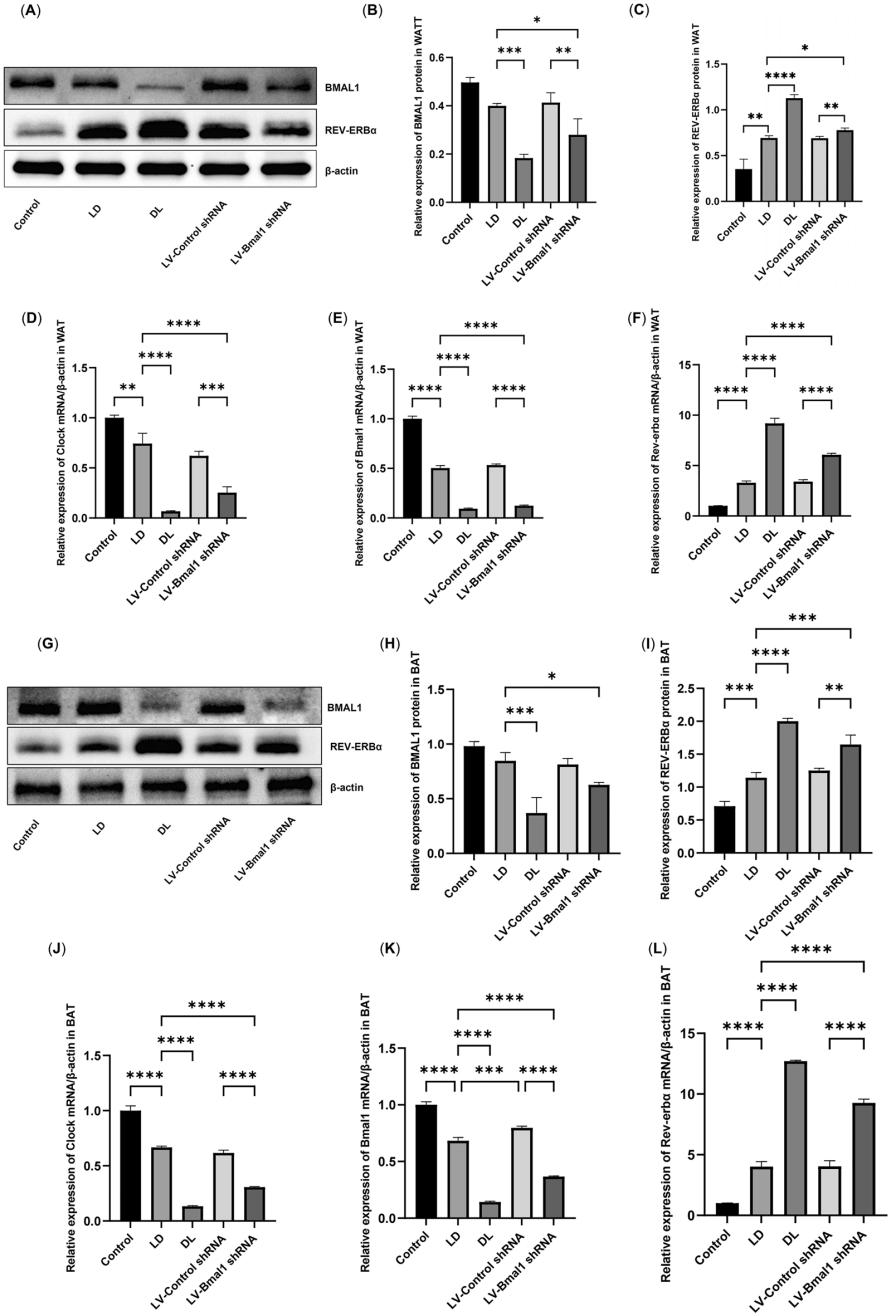
BMAL1/REV-ERBα Circadian Rhythmic Loop Disruption was observed in adipose tissue of HF. (**A**–**C**) Protein expression of BMAL1 and REV-ERBα measured by Western blot in WAT. (**D**–**F**) mRNA expression of Clock, Bmal1 and Rev-erbα measured by RT‒qPCR in WAT. (**G**–**I**) Protein expression of BMAL1 and REV-ERBα measured by Western blot in BAT. (**J**–**L**) mRNA expression of Clock, Bmal1 and Rev-erbα measured by RT‒qPCR in BAT. Data are mean ± SD, n = 6. *P < 0.05, **P < 0.01, ***P < 0.001, ****P < 0.0001.

We tested the expression of the central feedback loop of the biological clock (BMAL1 and REV-ERBα proteins) in the context of HF. According to our results, in both WAT and BAT, compared with healthy rats in the control group, the protein expression of REV-ERBα was increased (Fig. 2A,C,G,I, P < 0.01), and BMAL1 was suppressed in the LD group, although there was no significant difference (Fig. 2A,B,G,H, P > 0.01). Consistently, the mRNA levels of Clock and Bmal1 in both WAT and BAT of rats treated with MCT were decreased, while the mRNA levels of Rev-erbα were increased compared to those in healthy rats in the control group (Fig. 2D–F,J–L, P < 0.01). As expected, compared with HF rats housed in a normal diurnal cycle (LD group), suppression of Bmal1 expression via injection of LV-Bmal1 shRNA (LV-Bmal1 shRNA group) led to a suppression in the mRNA and protein levels of Clock and Bmal1, while upregulating Rev-erbα expression (P < 0.05).

### Disruption of the BMAL1/REV-ERBα circadian rhythmic loop resulted in adipose loss and ectopic lipid deposition in HF

**Figure 3:**
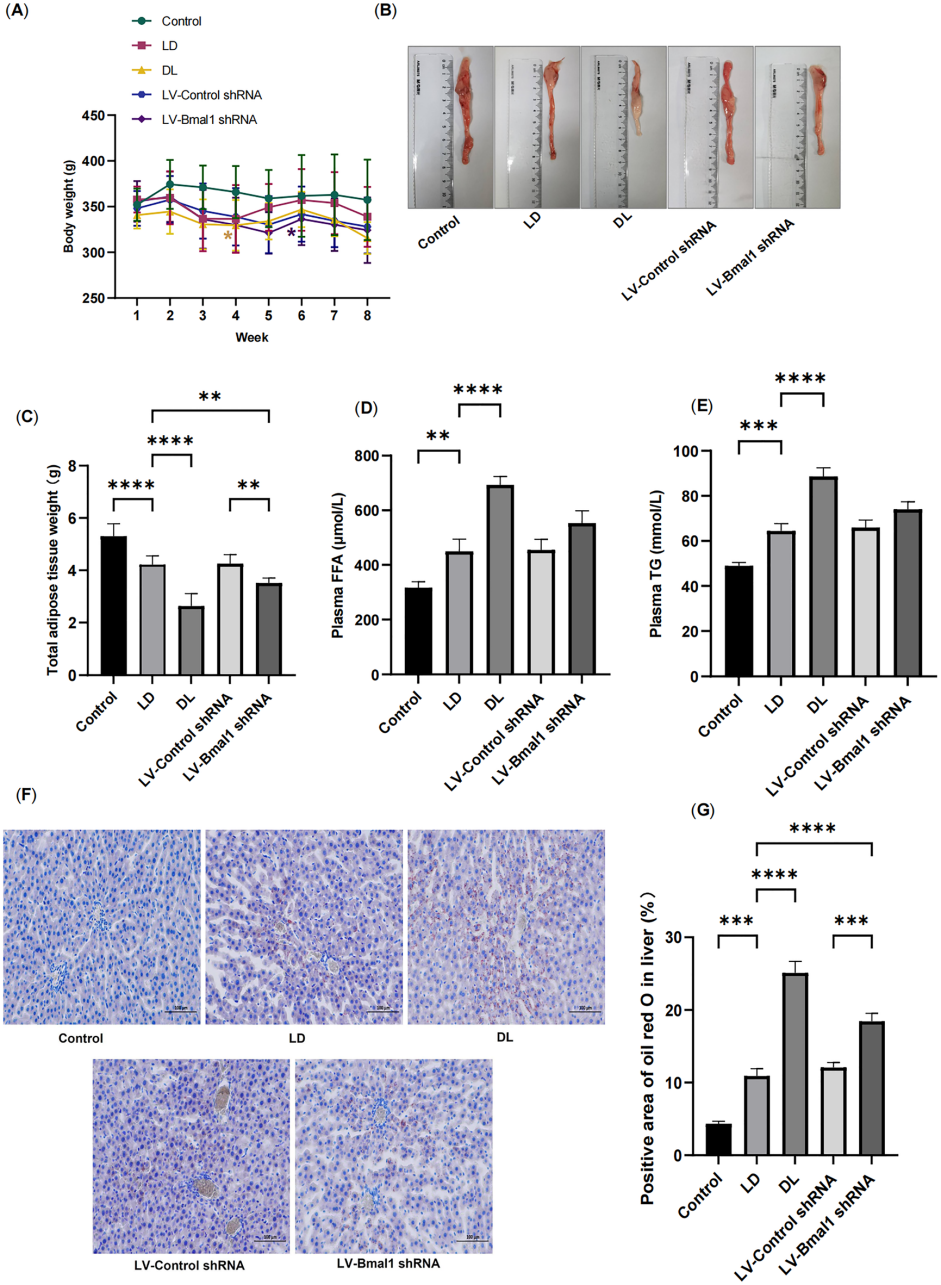
Disruption of the BMAL1/REV-ERBα circadian rhythmic loop resulted in body weight loss and total fat mass and caused increased lipid ectopic deposition in HF. (**A**) Changes in body weight through the duration of the 8-week study. (**B**) Representative image of epididymal fat volume. (**C**) Total adipose tissue weight. (**D**) Plasma FFA levels. (**E**) Plasma TG levels. (**F**,**G**) Representative images of Oil O staining and positive area of liver tissue. Data are mean ± SD, n = 6. *P < 0.05, **P < 0.01, ***P < 0.001, ****P < 0.0001.

During the experimental period, rats that were administered MCT in the LD group exhibited reduced body weight after 2 weeks of MCT injection compared with rats that were not administered MCT in the control group, although there was no significant difference. Rats in both the DL group and LV-Bmal1 shRNA group exhibited significant weight loss compared with rats in the control group at the 3rd week and 5th week, respectively (Fig. 3A, P < 0.05). At the 8th week, we evaluated total adipose tissue weight. As expected, rats in the LD group exhibited a significant decrease compared with rats in the control group (Fig. 3B,C, P < 0.01). Compared with rats in the LD group, rats housed in a disrupted light: dark cycle and injected with LV-Bmal1 shRNA exhibited more severe adipose loss (P < 0.01).

As described previously, dyslipidaemia and fatty liver attributed to FFAs released from excessive lipolysis deposits in ectopic tissue^27^. In the present study, we found that MCT-injected rats housed in the light: dark cycle exhibited increased blood FFA and TG levels compared with rats in the control group; importantly, reversal of the light–dark cycle resulted in higher blood levels of both FFA and TG in the DL group (Fig. 3D,E, P < 0.01). Consistently, rats in the LD group exhibited increased lipid deposition in the liver compared with healthy rats in the control group (Fig. 3F,G, P < 0.01); moreover, rats in both the DL group and LV-Bmal1 shRNA group exhibited more lipid deposition than rats in the LD group (P < 0.01).

### Disruption of the BMAL1/REV-ERBα circadian rhythmic loop resulted in adipocyte atrophy and increased extracellular matrix in adipose tissue under conditions of HF

**Figure 4:**
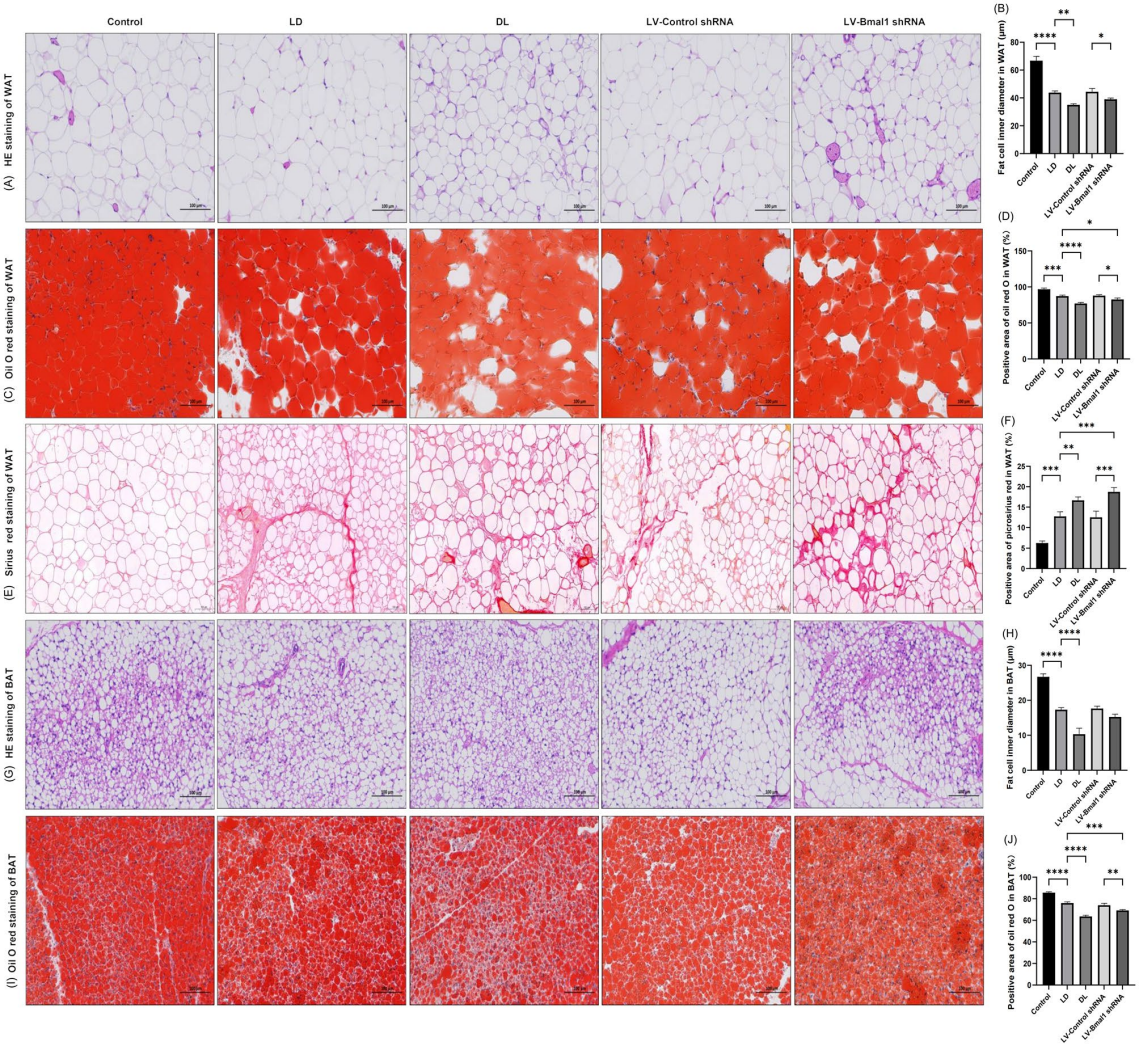
Disruption of the BMAL1/REV-ERBα circadian rhythmic loop resulted in adipocyte atrophy and increased extracellular matrix in adipose tissue under conditions of HF. (**A**,**B**) Representative images of HE staining and fat cell inner diameter of WAT. (**C**,**D**) Representative images of Oil O staining and positive area of WAT. (**E**,**F**) Representative images of picrosirius red staining and positive area of WAT. (**G**,**H**) Representative images of HE staining and fat cell inner diameter of BAT. (**I**,**J**) Representative images of Oil O staining and positive area of BAT. Data are mean ± SD, n = 3. *P < 0.05, **P < 0.01, ***P < 0.001, ****P < 0.0001.

Using HE staining and Oil Red O staining, we observed that rats treated with MCT in the LD group exhibited smaller adipocytes (Fig. 4A,B,G,H) and decreased lipid accumulation (Fig. 4C,D,I,J) in both WAT and BAT compared with rats in the control group (P < 0.01). This observation was more significant when the light–dark cycle was reversed or when lentiviral injection suppressed Bmal1 gene expression, with rats in both the DL group and LV-Bmal1 shRNA group having less lipid accumulation than MCT-injected rats housed in normal circadian rhythm, as shown by Oil Red O staining (P < 0.01). Moreover, rats in the DL group had smaller adipocytes in both WAT and BAT than rats in the LD group (P < 0.01). Additionally, we revealed that there was increased extracellular matrix deposition in the WAT of rats in the LD group after induction of cardiac cachexia by injection of MCT compared with rats in the control group (Fig. 4E,F, P < 0.01). Moreover, reversing the circadian cycle or lentiviral inhibition of Bmal1 further aggravated extracellular matrix deposition (P < 0.01).

### Disruption of the BMAL1/REV-ERBα circadian rhythmic loop resulted in lipolysis and Beiging of WAT and reducing lipid storage in HF

**Figure 5:**
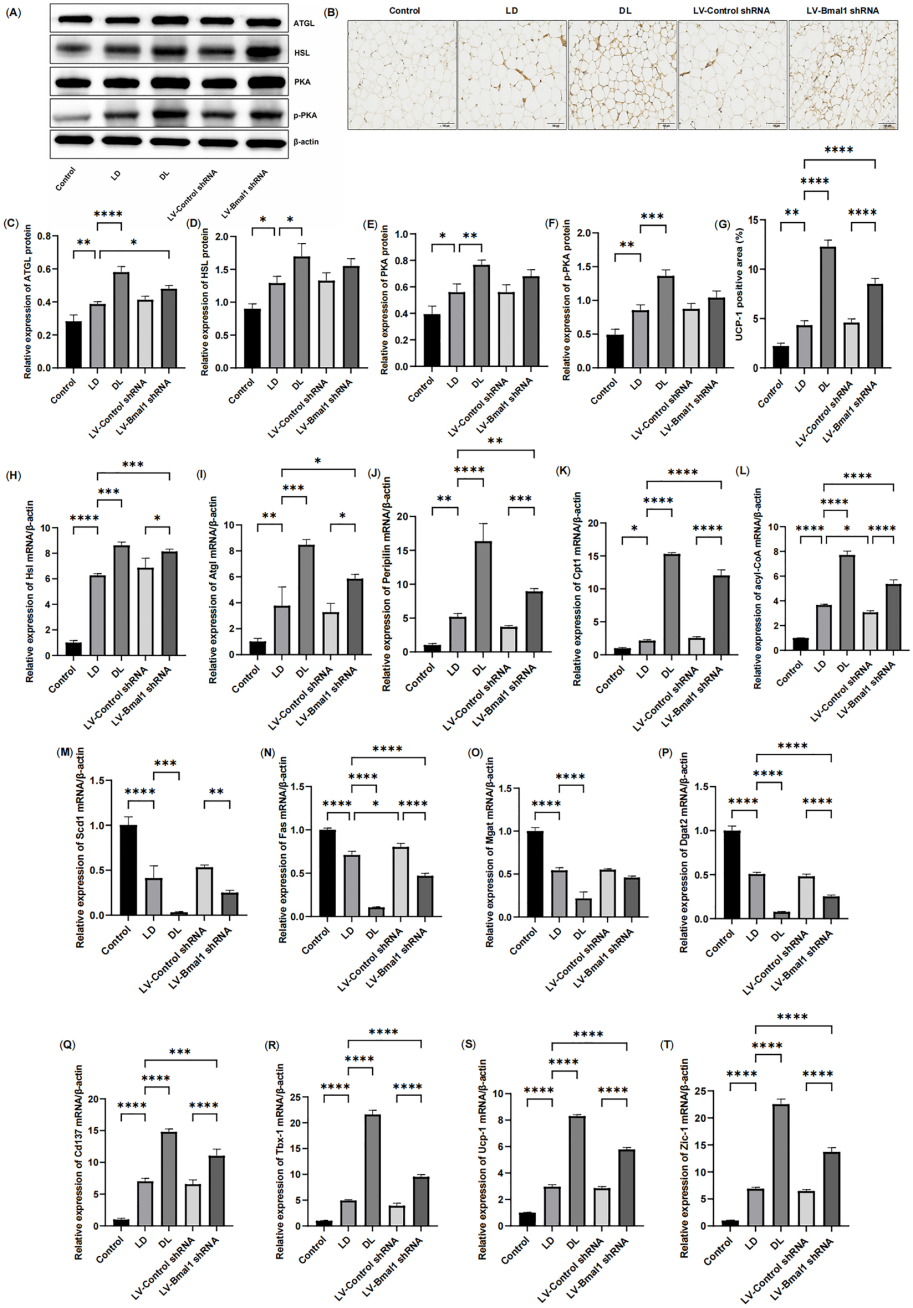
Disruption of the BMAL1/REV-ERBα circadian rhythmic loop resulted in lipolysis and beiging of WAT and reducing lipid storage in HF. (**A**,**C**–**F**) Protein expression of ATGL, HSL, PKA and p-PKA measured by Western blot in WAT. (**B**,**G**) Immunostaining and positive areas of UCP-1 in WAT. (H-J) mRNA expression of Hsl, Atgl and Peripilin in WAT. (**K**,**L**) mRNA expression of FFA β-oxidation genes, including Cpt1 and acyl-CoA. (**M**–**P**) mRNA expression of lipogenic genes, including Scd1, Fas, Mgat and Dgat2. (**Q**–**T**) mRNA expression of beige adipocyte markers, including Cd137, Tbx-1, Ucp-1 and Zic-1. Data are mean ± SD, n = 6. *P < 0.05, **P < 0.01, ***P < 0.001, ****P < 0.0001.

To test the effect of circadian rhythm disorder on WAT lipolysis in HF, we measured the protein expression of protein lipase (PKA) signalling and its downstream enzymes primarily responsible for the hydrolysis of lipid droplets, including ATGL and HSL. According to our results, we found that the expression of ATGL, HSL, PKA and p-PKA increased under conditions of HF compared with non-HF rats (Fig. 5A,C–F, P < 0.01, P < 0.05). These results were more obviously due to injection of LV-Bmal1 shRNA, with rats in the LV-Bmal1 shRNA group exhibited higher protein levels of ATGL, HSL, PKA and p-PKA, although only ATGL was significantly different (P < 0.05). Consistently, compared with the control group, the mRNA expression levels of lipase genes (Hsl, Atgl and Peripilin) (Fig. 5H–J) and FFA β-oxidation genes (Cpt1 and acyl-CoA) (Fig. 5K,L) increased under conditions of HF (P < 0.05). These genes exhibited higher expression levels in the rats that experienced injection of LV-Bmal1 shRNA compared to those injected with the empty lentiviral vehicle (P < 0.01, P < 0.05).

A previous study suggested that clock genes are involved in adipogenesis by directly and indirectly regulating transcription factors involved in lipogenesis processes^28^. In the present study, we found that the expression of the lipogenic genes Scd1, Fas, Mgat and Dgat2 decreased in WAT of HF compared with controls (Fig. 5M–P, P < 0.01). Lentiviral inhibition of Bmal1 resulted in further decreased lipogenesis in white adipocytes, reflected by decreased mRNA expression of Scd1, Fas, Dgat2, and Mgat in the LV-Bmal1 shRNA group.

Beiging of WAT is regarded as a driver of fat expenditure in cachexia^29^. As described previously in cancer-associated cachexia^30^, we found that beige adipocyte markers, including the mRNA expression of Cd137, Tbx-1, Ucp-1 and Zic-1 (Fig. 5Q–T, P < 0.01) as well as the protein expression of UCP-1 (Fig. 5B,G, P < 0.01), were increased in LD group compared with those in rats without HF. Lentiviral inhibition of Bmal1 aggravated beiging of WAT, as shown by the increased protein expression of UCP-1 and mRNA expression of Cd137, Tbx-1, Ucp-1, and Zic-1 in the LV-Bmal1 shRNA group compared with the LD group (P < 0.01).

### Disruption of the BMAL1/REV-ERBα circadian rhythmic loop resulted in PKA-mediated thermogenesis in BAT in HF

**Figure 6:**
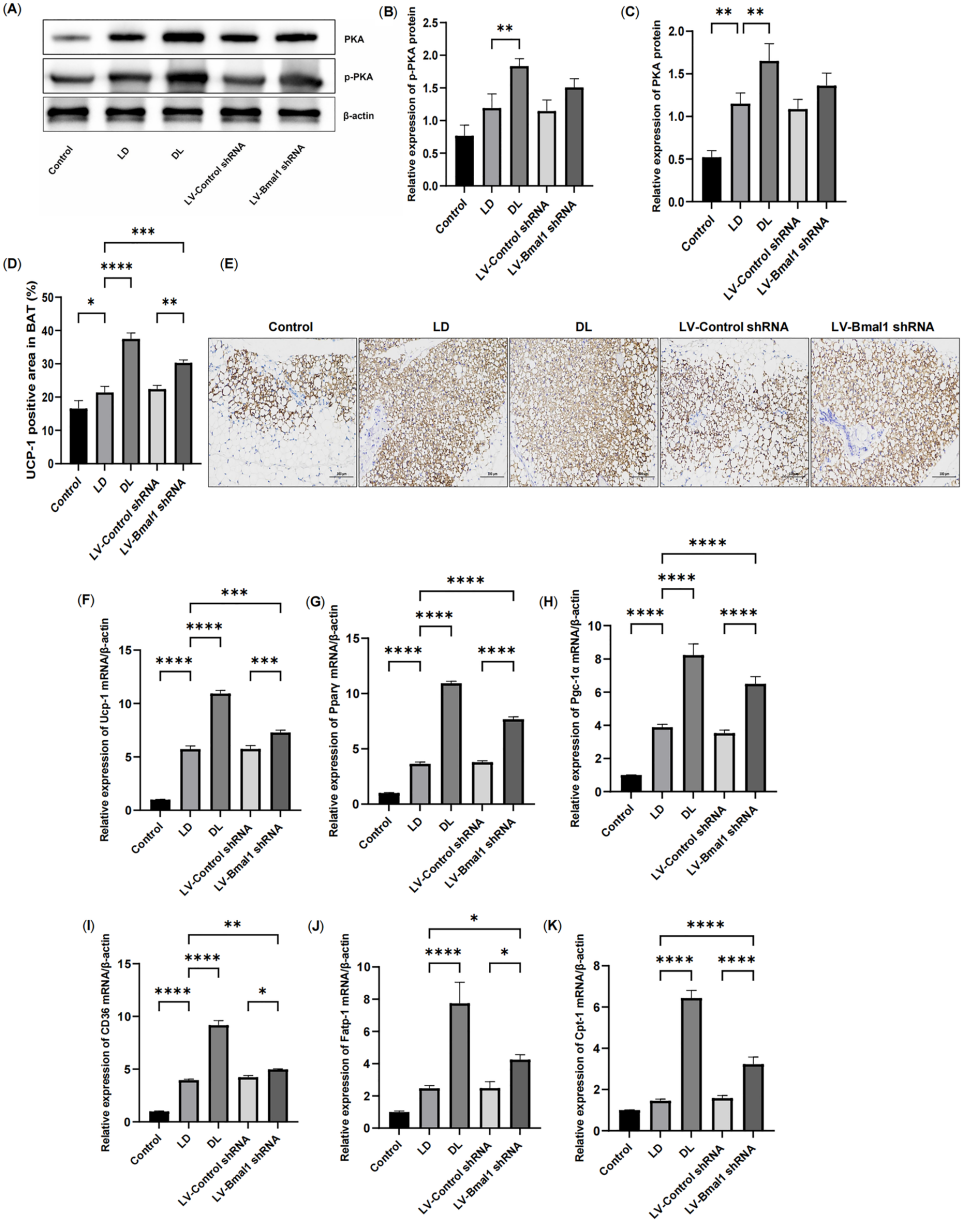
Disruption of the BMAL1/REV-ERBα circadian rhythmic loop resulted in PKA-mediated thermogenesis in BAT in HF. (**A**–**C**) Protein expression of PKA and p-PKA measured by Western blot in BAT. (**D**, **E**) Immunostaining and positive areas of UCP-1 in BAT. (**F**–**H**) mRNA expression of thermogenesis genes, including Ucp-1, Pparγ and Pgc-1α. (**I**–**K**) mRNA expression of lipid transportation genes, including Cd36, Fatp-1 and Cpt-1. Data are mean ± SD, n = 6. *P < 0.05, **P < 0.01, ***P < 0.001, ****P < 0.0001.

In brown adipocytes, activated PKA signalling initiates thermogenesis by upregulating thermogenesis genes, such as Pparγ and Pgc-1α to induce UPC-1 expression. During this process, FFAs derived from white adipocyte lipolysis are transported into brown adipocytes by transport proteins such as fatty acid transport protein (FATP) and CD36 and then transported to mitochondria via CPT-1 to serve as substrates for mitochondrial thermogenesis^25^. As described previously, BAT exhibits elevated thermogenesis in cancer-induced cachexia^31^. In our present study, compared with healthy rats, we found that HF exhibited elevated thermogenesis in brown adipocytes exhibiting increased protein levels of PKA, p-PKA and UCP-1 (Fig. 6A–E, P < 0.01, P < 0.05), although p-PKA had no significant difference. Meanwhile, thermogenesis genes (Ucp-1, Pparγ and Pgc-1α) and lipid transportation genes (Cd36, Fatp-1, Cpt-1) increased (Fig. 6F–I,K, P < 0.01). Similarly, suppressing the key clock gene Bmal1 also increased UCP-1 protein levels and upregulated thermogenesis genes (Ucp-1, Pparγ and Pgc-1α) and lipid transportation genes (Cd36, Fatp-1 and Cpt-1). Altogether, our study showed activated thermogenesis in BAT in conditions of HF, contributing to enhanced adipose expenditure. More importantly, lentiviral inhibition of Bmal1 aggravated thermogenesis in BAT.

### Disruption of the BMAL1/REV-ERBα circadian rhythmic loop resulted in elevated factors contributing to fat depletion, including NT-proBNP, IL-6 and NE

**Figure 7:**
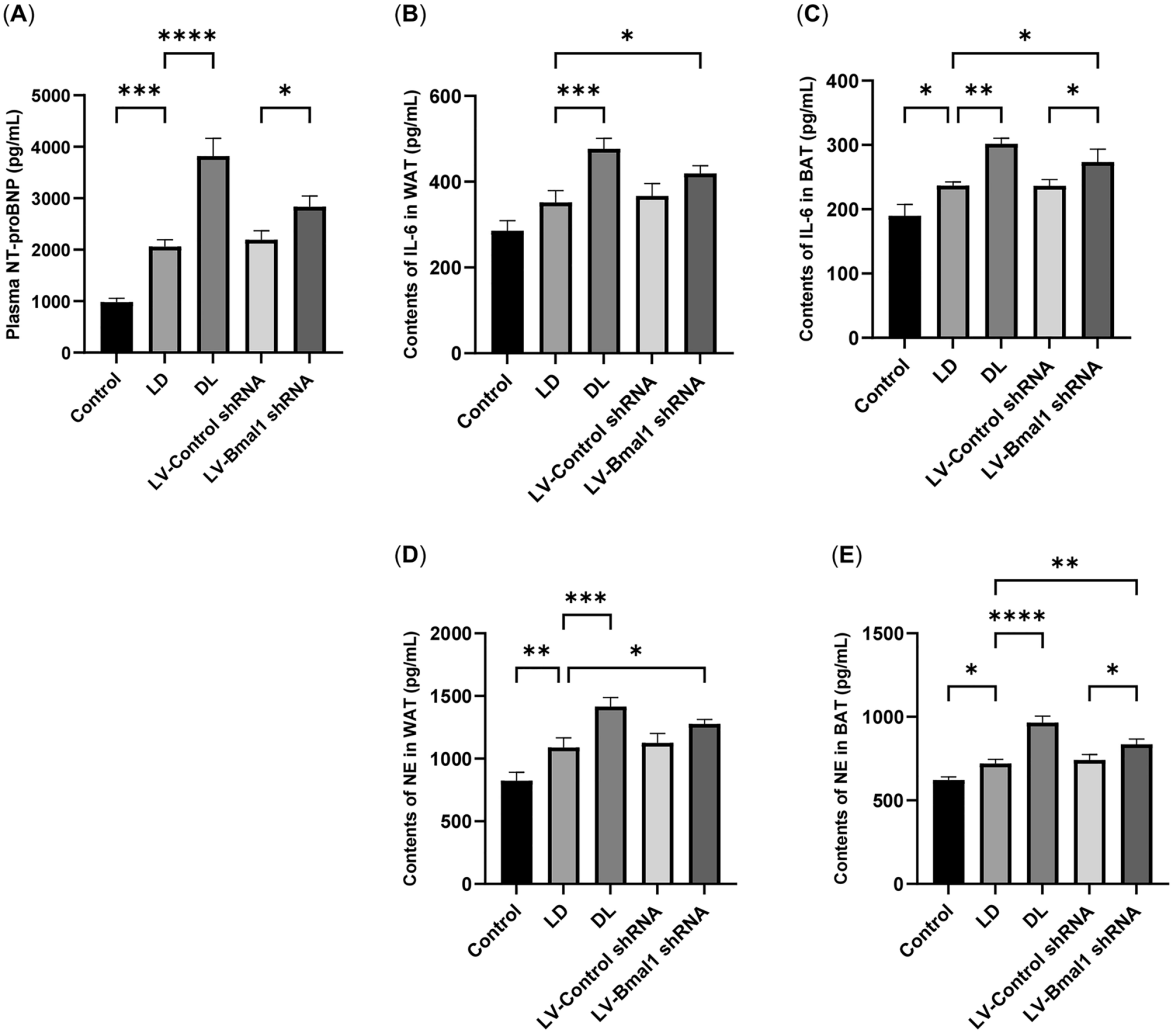
Disruption of the BMAL1/REV-ERBα circadian rhythmic loop resulted in elevated factors contributing to fat depletion, including NT-proBNP, IL-6 and NE. (**A**) Plasma levels of NT-proBNP. (**B**) IL-6 content in WAT. (**C**) IL-6 content in BAT. (**D**) NE content in WAT. (**E**) NE content in BAT. Data are mean ± SD, n = 6. *P < 0.05, **P < 0.01, ***P < 0.001, ****P < 0.0001.

Studies have suggested that increased NT-proBNP, IL-6 and NE contribute to the prevalence of adipose depletion in HF^32–34^. In our study, we observed higher levels of NT-proBNP in blood (Fig. 7A, P < 0.01) and higher levels of IL-6 and NE in adipose tissue (Fig. 7B–E, P < 0.01, P < 0.05) in the LD group of rats treated with MCT compared with the control group. Injecting LV-Bmal1 shRNA further elevates the blood levels of NT-proBNP and adipose tissue levels of IL-6 and NE.

## Discussion

Adipose tissue is integral to maintaining energy balance and regulating lipid metabolism. Dysregulation of adipose function is closely associated with metabolic disorders such as obesity and cachexia^35^, including the development of cardiac cachexia through adipose depletion^32^. However, the molecular mechanisms governing fat expenditure in HF remain poorly understood. In this study, we investigated the impact of disrupting the BMAL1/REV-ERBα circadian rhythmic loop on fat expenditure in HF.

Emerging evidence indicates that the circadian clock influences lipid metabolism and is closely associated with metabolic disorders^10^. However, the specific link between the circadian clock and fat expenditure remains unclear. Bmal1, a key transcription factor in the mammalian circadian clock, plays a crucial role in regulating circadian behaviors and metabolic functions^36,37^. Our study demonstrates decreased expression of Clock and Bmal1, along with increased levels of Rev-erbα in both WAT and BAT in HF. These findings suggest an imbalanced Clock:Bmal1/Rev-erbα circuitry contributing to fat expenditure in HF.

Fat droplets can account for 95% of the total volume of white adipocytes and are mainly composed of TGs. During lipolysis, TGs within these droplets undergo hydrolysis into FFAs, serving as fuel in peripheral tissues based on metabolic requirements. In cachexia-induced hypermetabolism, excessive TGs are hydrolyzed, leading to adipocyte atrophy and increased FFA ectopic deposition in peripheral tissues^25^. Similarly, our results revealed that MCT-induced HF exhibited increased ectopic lipid accumulation reflected by elevated blood levels of TG and FFA as well as excessive fat accumulation in the liver. Furthermore, HF rats exhibit reduced adipocyte size and lipid content in both WAT and BAT, as evidenced by HE and Oil Red O staining. Notably, altering the light–dark cycle and lentiviral inhibition of Bmal1 exacerbate adipocyte size reduction, lipid content decrease in fat droplets, and worsen ectopic lipid deposition during cachexia progression in the hypermetabolic state.

In obesity, persistent inflammation leads to an accumulation of extracellular matrix proteins in adipose tissue, impairing its ability to adapt to changing metabolic conditions^38^. Similarly, in cancer cachexia, adipose tissue exhibits fibrotic areas with increased expression of extracellular matrix components^39^. Our findings consistently show elevated extracellular matrix deposition in HF rats. Importantly, disrupting the circadian clock, either by altering the diurnal cycle or downregulating Bmal1 expression, exacerbates fibrosis in WAT. This suggests a significant role for circadian rhythm disruption in promoting extracellular matrix accumulation, particularly in the context of HF-induced cachexia.

WAT stores energy as TGs during high-energy periods and releases them as fuel when needed^9^. FFA release from TGs is primarily mediated by PKA, which phosphorylates enzymes like perilipin and HSL, indirectly affecting ATGL^27^. FFAs produced are transported into mitochondria for beta-oxidation, controlled by enzymes like Cpt-1 and acyl-CoA^40^. As key enzymes of lipolysis, HSL and ATGL play a critical role in the process of cancer cachexia. In patients with cachexia, the activities of HSL and ATGL are increased^41^. Depletion of ATGL and HSL in a murine cancer cachexia model protects against white adipose tissue loss, with HSL knockout providing less protection^41^. Adipocyte clocks regulate lipolysis by controlling the transcription of lipolysis enzymes in a circadian manner, with CLOCK:BMAL1 heterodimers regulating ATGL and HSL transcription. Disruption of circadian rhythms, as seen in Clock∆19 and Bmal1^−/−^ mutant mice, disrupts circadian variations in ATGL and HSL expression^20^. In our study, PKA signaling-mediated lipid droplet hydrolysis was markedly stimulated in HF. Remarkably, lentiviral inhibition of Bmal1 significantly increased lipolysis and FFA β-oxidation in white adipocytes, evidenced by upregulated protein expression of PKA, p-PKA, ATGL, and HSL, and increased mRNA levels of Atgl, Hsl, perilipin, Cpt1, and acyl-CoA. These findings suggest that disruption of the BMAL1/REV-ERBα circadian loop contributes significantly to promoting lipolysis in HF.

Adipocytes synthesize FFAs from carbohydrates through de novo lipogenesis, contributing to TG storage in lipid droplets. DGAT2, FAS, and SCD1 are key enzymes involved in TG synthesis, while MGAT promotes TG synthesis by interacting with DGAT^42,43^. Several studies have suggested a critical function of clock genes, especially BMAL1, in regulating lipid synthesis and storage in white adipocytes^19,44,45^. Depletion of Bmal1 reduces lipogenesis genes (Acc1, Fas, Scd1, Gpat) in the liver, while Bmal1 overexpression increases mRNA expression of lipogenic enzymes^46^. Our study observed reduced lipogenesis and lipid storage in white adipocytes of HF rats, further exacerbated when downregulating Bmal1 expression disrupted the BMAL1/REV-ERBα circadian rhythmic loop. Thus, we speculated that an imbalanced circadian clock interferes with lipogenesis and lipid storage in white adipocytes by suppressing lipogenesis genes, which is an unfavourable factor for lipid storage in condition of HF.

Enlarged beiging of WAT occurs in conditions of hypermetabolism like severe burn injury and cancer, converting stored lipids into heat via thermogenesis, leading to increased fat loss. Recently, limited studies have reported beiging adipocytes in WAT of HF animals^47,48^. In our study, we observed increased beiging markers, including Ucp-1, Cd137, Tbx-1, and Zic-1, indicating heightened beiging of WAT in HF induced by MCT injection. This was consistent with our previous study using a salt-sensitive hypertension-induced HF rat model^25^. Additionally, our study is the first to demonstrate that disruption of the BMAL1/REV-ERBα circadian rhythmic loop induces beiging of WAT in HF.

**Figure 8:**
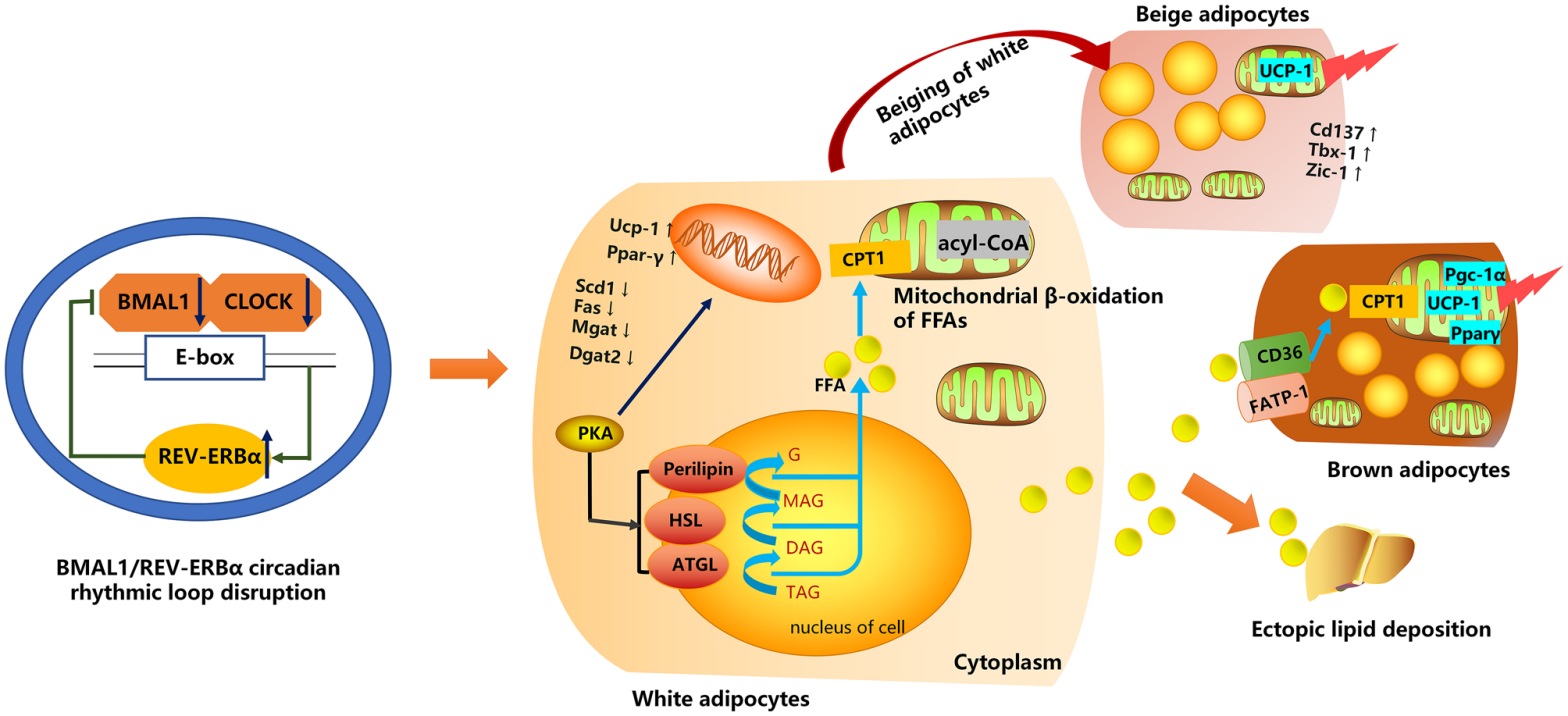
Mechanism of the BMAL1/REV-ERBα circadian rhythmic loop disruption in HF leading to fat expenditure. Disruption of the BMAL1/REV-ERBα circadian rhythmic loop leads to lipolysis of WAT, thermogenesis in beige/brown adipocytes, and inhibition of WAT production and storage, which is associated with excessive fat expenditure in HF.

BAT is a metabolically active organ known for its unique ability to undergo adaptive thermogenesis in response to cold and adrenergic stimuli^49^. This process involves the conversion of energy into heat through β-oxidation or uncoupling of mitochondrial proton transport via UCP-1^50^. Upon stimulation by NE released from sympathetic nerve terminals, β3-adrenergic receptors on brown adipocytes activate the PKA pathway, leading to the upregulation of thermogenic genes such as Ucp-1, Pparγ, and Pgc-1α. Concurrently, this pathway activates ATGL, promoting the production of FFAs, which are then transported into brown adipocytes through proteins like CD36 and FATP-1. Subsequently, FFAs enter mitochondria via CPT, where they serve as substrates for mitochondrial thermogenesis^27^. Similar to WAT, the lipid metabolism and thermogenesis in BAT are influenced by the circadian clock^51^. Research by van den Berg et al.^52^ revealed a daily rhythm in FFA uptake by BAT, synchronized with the light/dark cycle and peaking upon waking. BMAL1 is crucial for lipid metabolism in BAT, as demonstrated in mice lacking Bmal1 in brown adipocytes, which exhibited dysregulated lipid metabolism and thermogenesis-related genes^53^. Rev-erbα plays a role in promoting brown adipogenesis and thermogenesis by upregulating UCP-1 expression^54,55^. In tumor-induced cachexia, disrupted diurnal expression patterns of lipid metabolism and thermogenesis pathways in brown adipocytes may contribute to fat expenditure^31^. However, the direct causal relationship between the circadian clock and increased fat expenditure via BAT thermogenesis remains unexplored. Our study unveils, for the first time, the role of BMAL1/REV-ERBα circadian rhythmic loop disruption in enhancing thermogenesis in BAT, thereby promoting adipose depletion in HF. As depicted in Fig. 8, our findings illustrate that disrupting the BMAL1/REV-ERBα circadian loop leads to WAT lipolysis, beige/brown adipocyte thermogenesis, and inhibition of WAT production and storage under HF conditions.

Additionally, sympathetic nervous system activation and inflammation are key upstream mechanisms in adipose tissue expenditure during cachexia^3^. Patients with chronic HF developing cachexia often exhibit elevated catecholamine levels^56^. The circadian clock regulates rhythmic catecholamine synthesis and secretion, potentially influencing cellular targets expressing adrenergic receptors^57^. Prolonged daylight reduces sympathetic input into BAT, impairing BAT activity by diminishing β3-adrenergic intracellular signaling^58^. Furthermore, chronic inflammation activation characterizes HF. The proinflammatory cytokine IL-6 upregulates UCP-1 expression, mediating beiging/browning thermogenesis, while suppressing lipid synthesis and accelerating lipolysis in white adipocytes^59,60^. Circadian clock dysregulation is associated with metabolic inflammation in adipose tissue, with the dark–light cycle exacerbating the IL-6-mediated inflammatory response^11,61^. NT-proBNP, a clinical marker reflecting HF severity, stimulates white adipocyte lipolysis and promotes beiging of white adipocytes, contributing to fat wasting in cachexia^32^. Although blood NT-proBNP levels exhibit diurnal rhythms, the direct connection between the circadian clock and NT-proBNP remains unclear^62^. Our study demonstrated elevated levels of NT-proBNP in blood, as well as NE and IL-6 in adipose tissue in MCT-induced HF rats. Additionally, inhibiting Bmal1 expression resulted in higher NT-proBNP levels in blood and elevated NE and IL-6 in adipose tissue. Thus, we deduced that the disruption of the BMAL1/REV-ERBα circadian rhythmic loop impaired diurnal variations in the synthesis and release of NT-proBNP, NE, and IL-6. These disruptions contributed to cachexia fat wasting by increasing lipolysis, decreasing lipid storage, and elevating beiging/BAT thermogenesis. Further studies are needed to elucidate detailed mechanisms linking adipose tissue expenditure and the circadian clock.

Furthermore, our findings suggest that HF induces a mild disruption in the BMAL1/REV-ERBα circadian rhythmic loop, potentially affecting the biological clock in rats. Prior research has indicated decreased expression of Bmal1 in the heart, liver, and kidney of rats with HF due to salt-sensitive hypertension compared to healthy controls^63^. Moreover, HF has been associated with insomnia, likely due to reduced melatonin levels crucial for maintaining circadian rhythms^64^. This HF-induced insomnia could further contribute to disruption of the biological clock. Our results only reveal tentatively that disruption of the BMAL1/REV-ERBα circadian rhythmic loop is associated with fat overconsumption in HF, and further studies are needed to demonstrate a causal relationship. Targeting the BMAL1/REV-ERBα circadian rhythmic loop for adipose tissue may serve as a novel target for the treatment of fat expenditure in HF. In addition, circadian-dependent therapeutic approaches such as "chronotherapy", "chronopharmacology" and "chrononutrition" should be considered when stabilising the biological clock.

## Limitation

There were several limitations in our study, firstly, we induced HF via injection of MCT, which caused a significant reduced fat mass as previously described^65^; however, we cannot excluded the toxicity of MCT in the present study. Secondly, food intake can influence body weight and fat weight. However, pair feeding was used during this study to minimize the effect of food intake. Therefore, statistical analysis of food intake was not performed. Moreover, the activity factors of the rats were not recorded. Lastly, it is necessary to explain that the DL group lacked a complete control because the sampling time could not be matched with the other groups, so the results of the DL group were interpreted only at the morphological level. Further study should observe the dynamic alteration of genes and proteins involved in lipid metabolism at different time points.

## Conclusion

In conclusion, our present study first provided evidence that the BMAL1/REV-ERBα circadian rhythmic loop disruption is associated with fat wasting in HF by promoting lipolysis, decreasing lipid storage in WAT and elevating beiging/brown adipocyte thermogenesis. We deduced that one of the molecular mechanisms underlying these results is attributed to the circadian clock directly or indirectly regulating the local transcriptome in a circadian manner, including key enzymes of lipogenesis, lipolysis and thermogenesis in both white and brown adipocytes. Additionally, the circadian clock controls the synthesis and release of NT-proBNP, NE and IL-6, which promote adipocyte lipolysis and beiging/brown adipocyte thermogenesis and is another molecular mechanism underlying excessive adipose tissue wasting in conditions of HF. From a clinical point of view, our study indicates that stabilizing adipose tissue rhythms may help to combat disrupted energy homeostasis and alleviate excessive adipose tissue expenditure in condition of HF.

### Supplementary Information

Supplementary Figures.

## Data Availability

The datasets supporting the conclusions of this article are included within the article.

## Abbreviations

HF: Heart failure
CLOCK: Circadian locomotor output cycles kaput
BMAL1: Brain and muscle aryl hydrocarbon receptor nuclear translocator-like protein 1
PER: Period
CRY: Cryptochrome
REV-ERB: Nuclear receptor reverse ERB
TGs: Triglycerides
ATGL: Adipose triglyceride lipase
MCT: Monocrotaline
RV-FAC: Right ventricular fractional area change
EDA: End-diastolic area
ESA: End-systolic area
WAT: White adipose tissue
BAT: Brown adipose tissue
IL-6: Interleukin-6
NE: Norepinephrine
Cpt1: Carnitine palmitoyl transferase 1
Scd1: Stearoyl-CoA desaturase 1
Fas: Fatty acid synthase
Mgat: Monoacylglycerol acyltransferase
Dgat2: Diacylglycerol acyltransferase-2
Zic-1: Zinc finger protein 1
Fatp-1: Fatty acid transport protein-1
Pparγ: Peroxisome proliferator-activated receptor gamma
Pgc-1α: Proliferator-activated receptor-γ coactivator 1α
PKA: Protein kinase A
p-PKA: Phospho-PKA
