# Supplementary material for: Excessive fat expenditure in MCT-induced heart failure rats is associated with BMAL1/REV-ERBα circadian rhythmic loop disruption
Source: Sci Rep. 2024 Apr 7;14:8128. doi: 10.1038/s41598-024-58577-8 (PMC10999456; doi:10.1038/s41598-024-58577-8)

Figure2-A

BMAL1

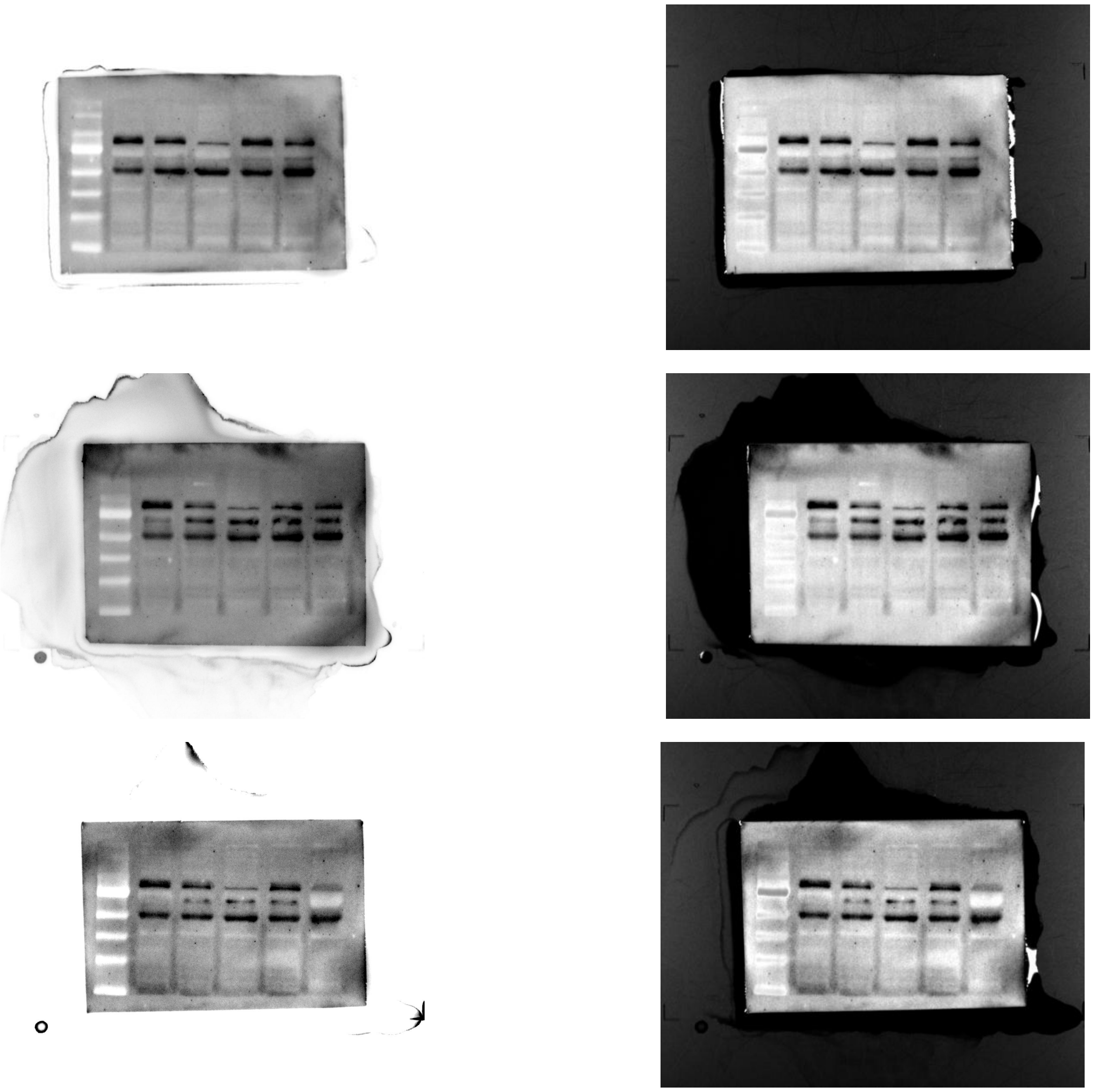

REV-ERB

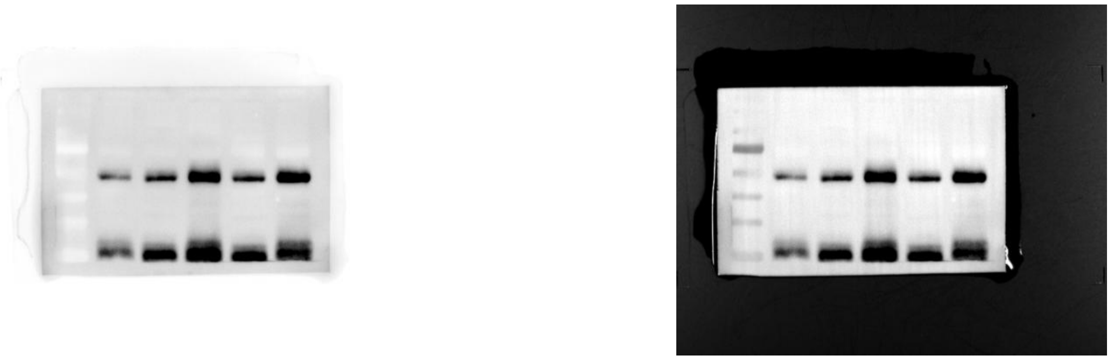

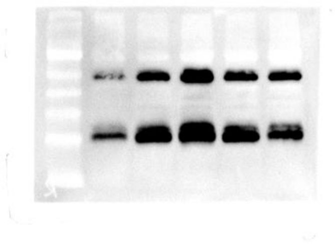

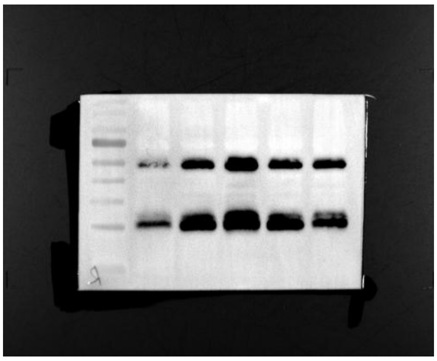

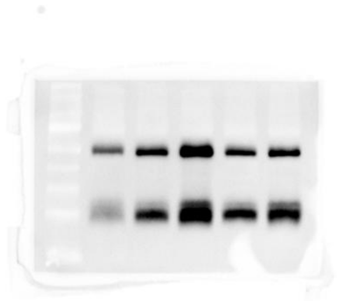

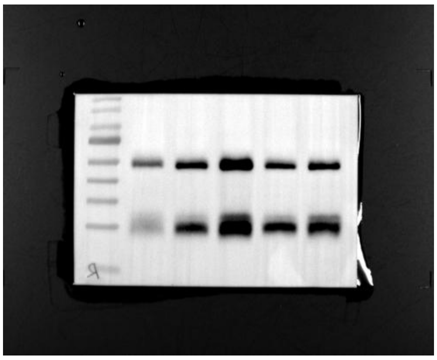

β-actin

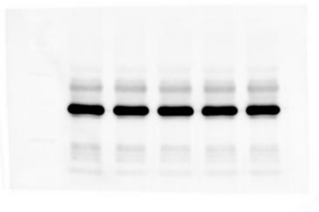

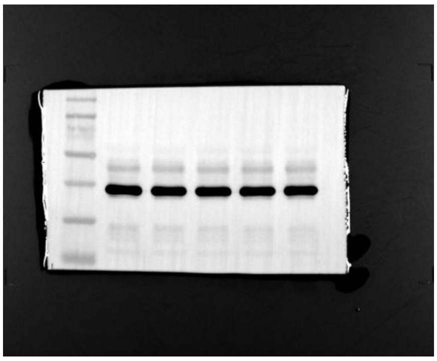

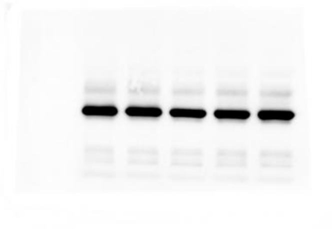

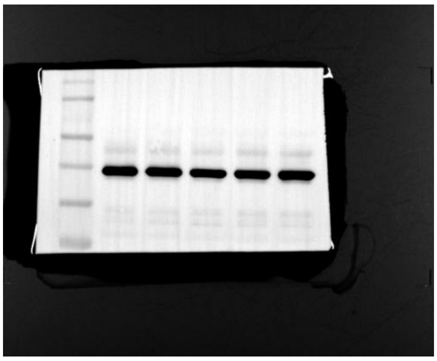

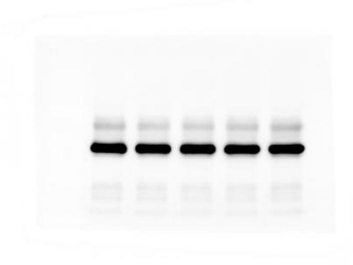
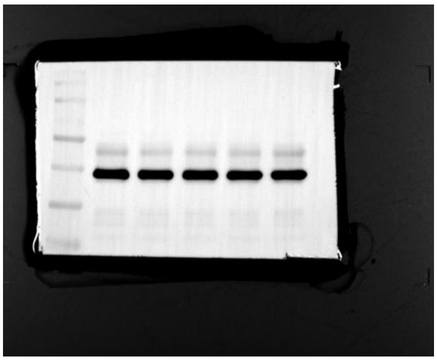

Figure2-G

BMAL1

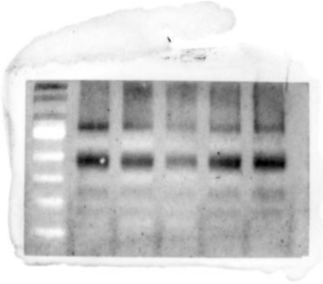
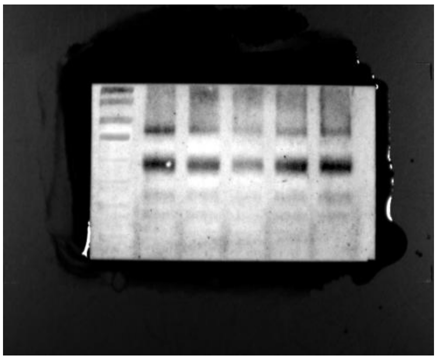
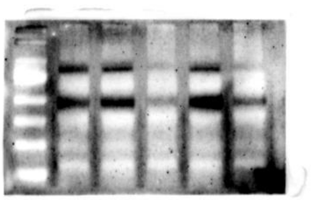
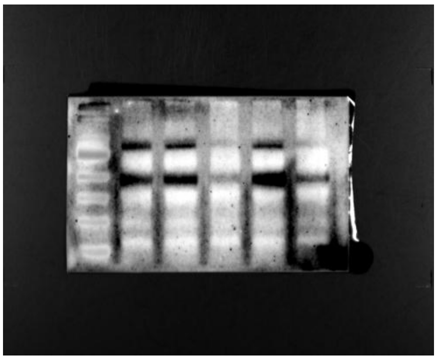
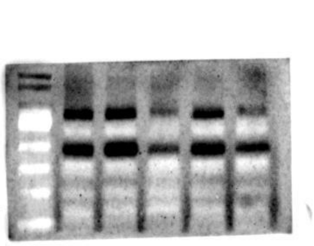
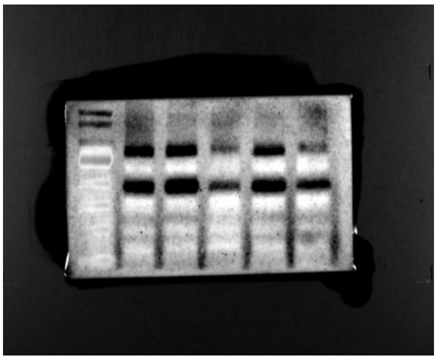

REV-ERB

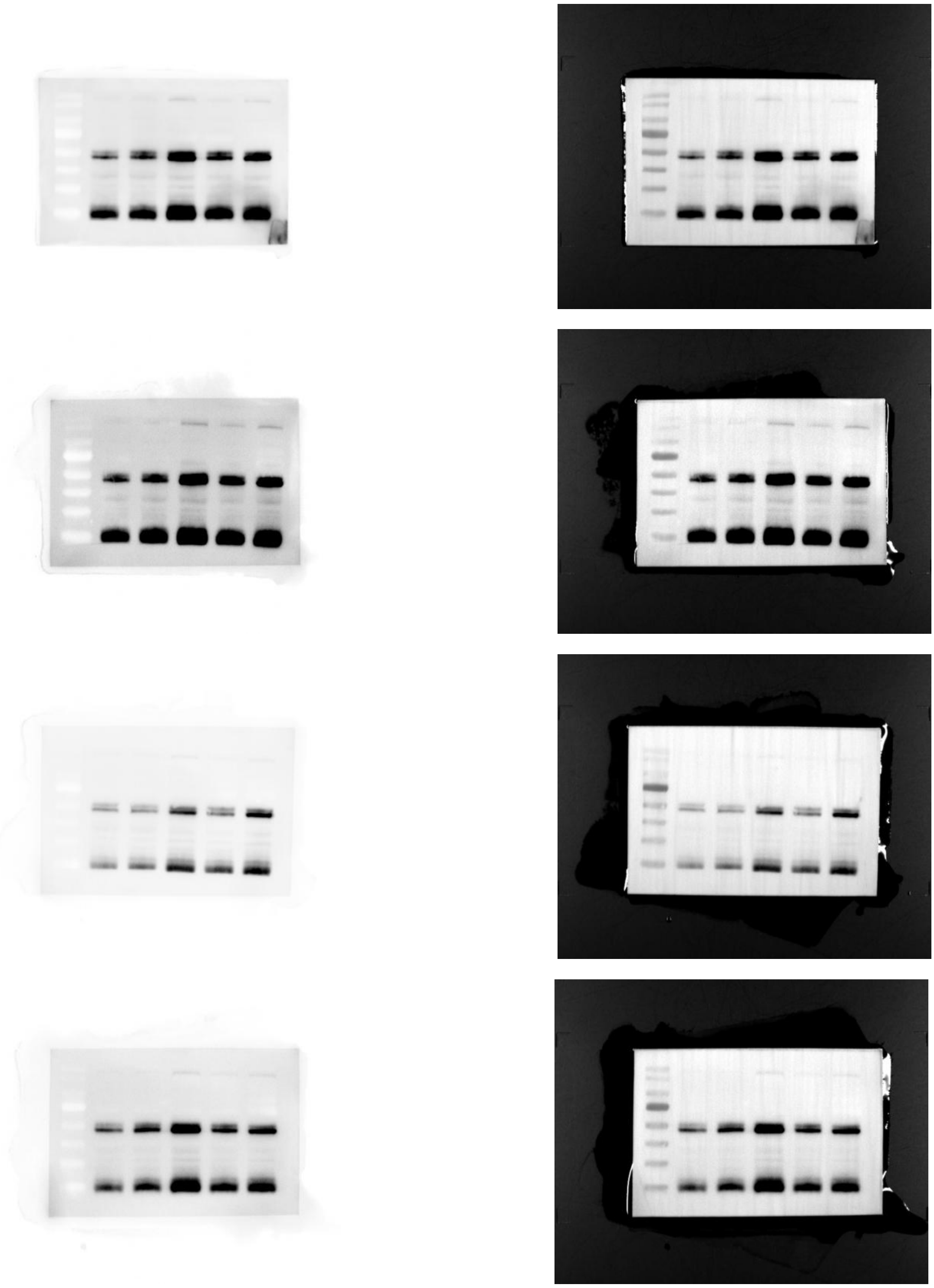

β-actin

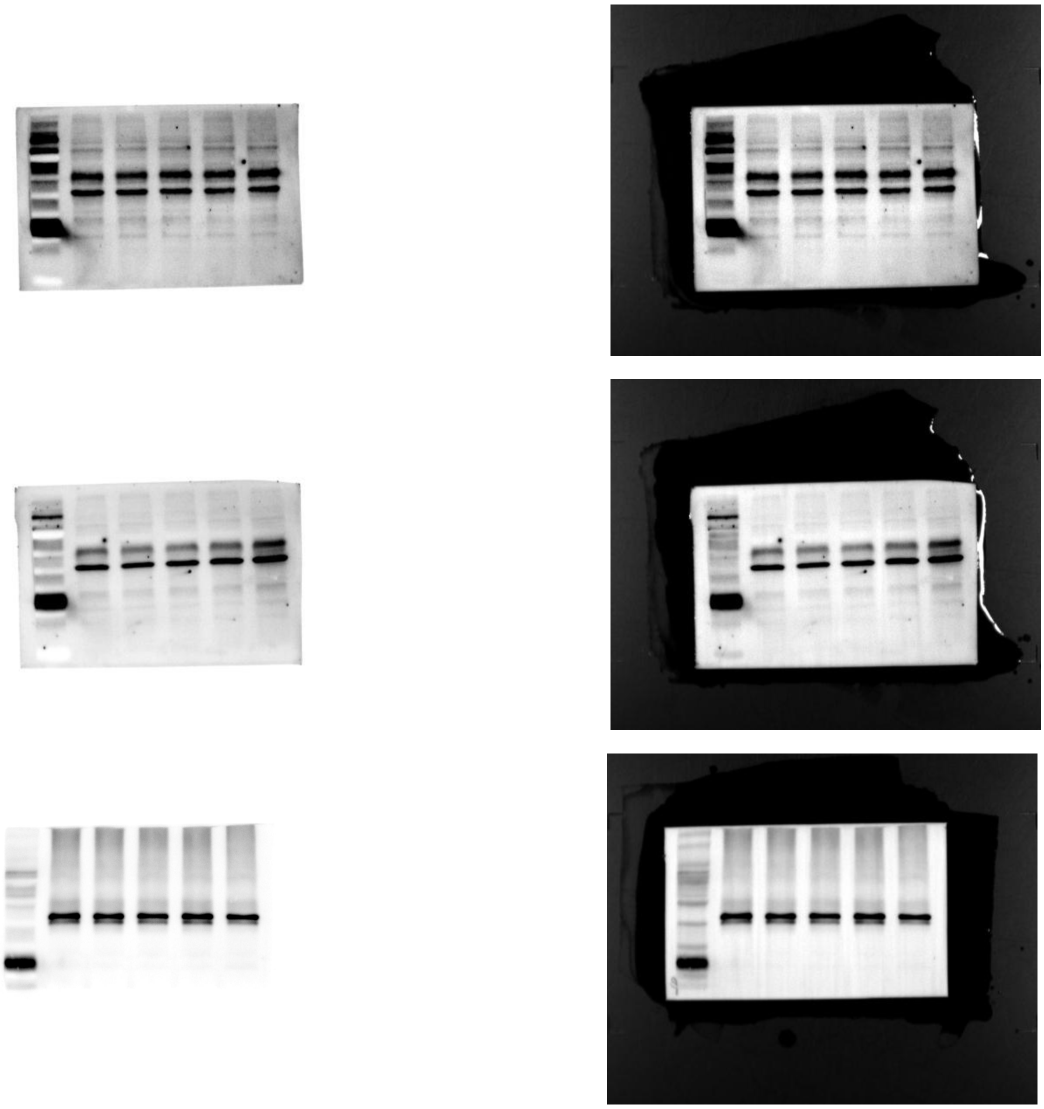

Figure5-A

ATGL

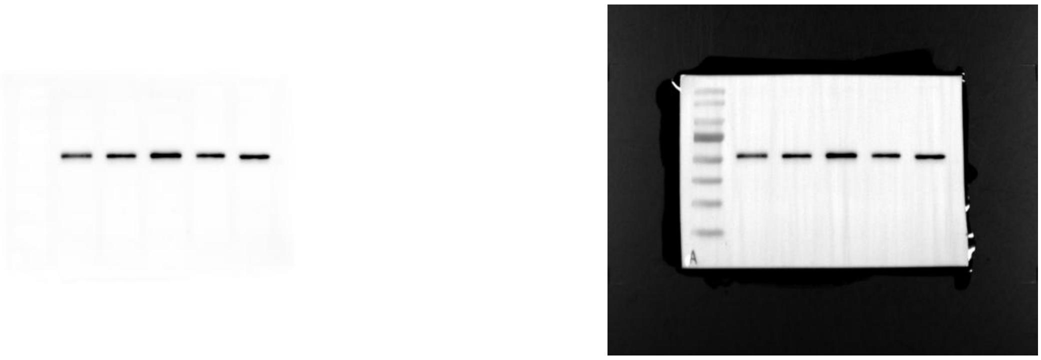

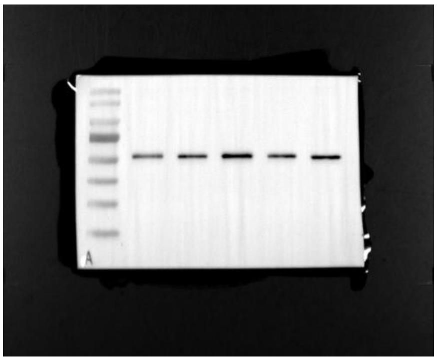

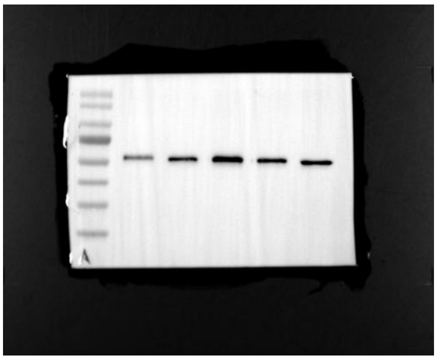
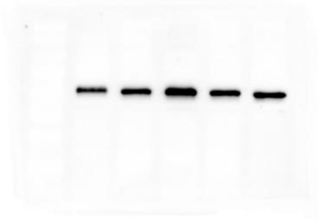
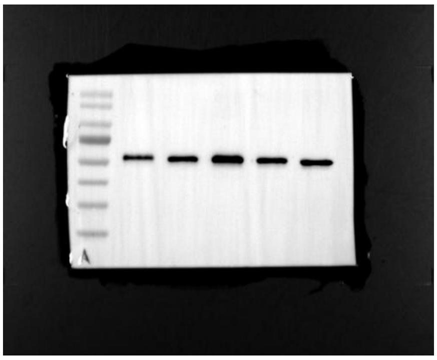

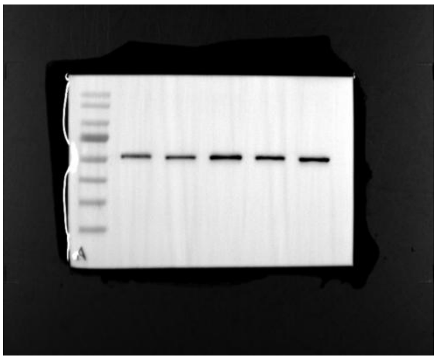
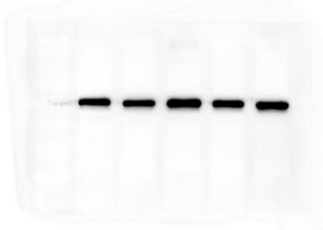
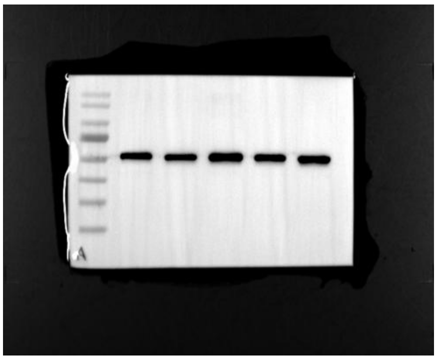

HSL

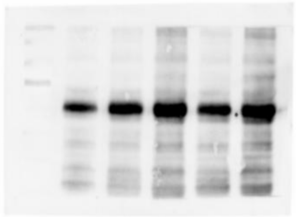

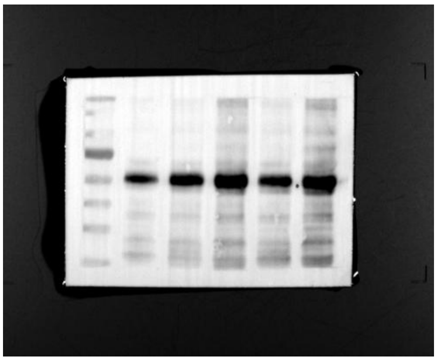

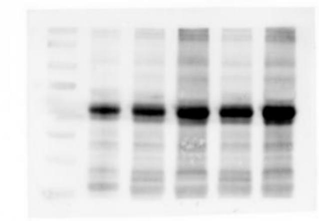

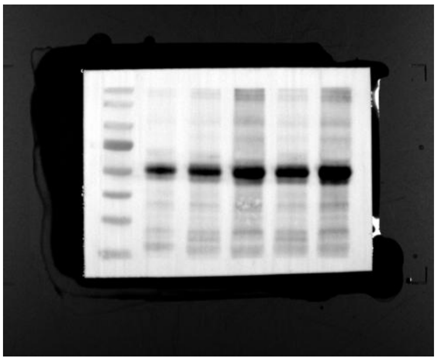

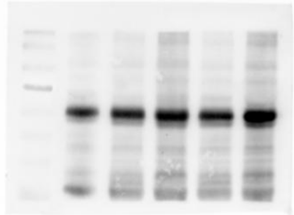

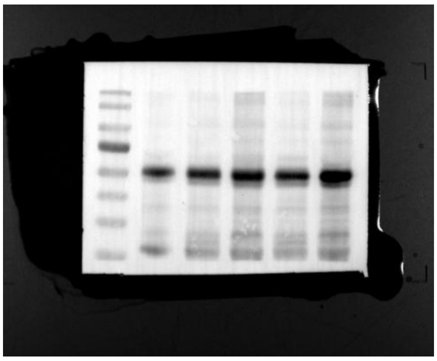

PKA

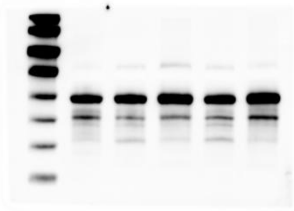

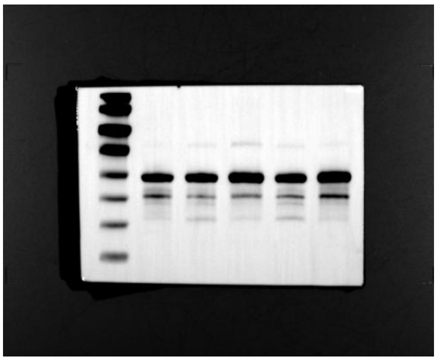

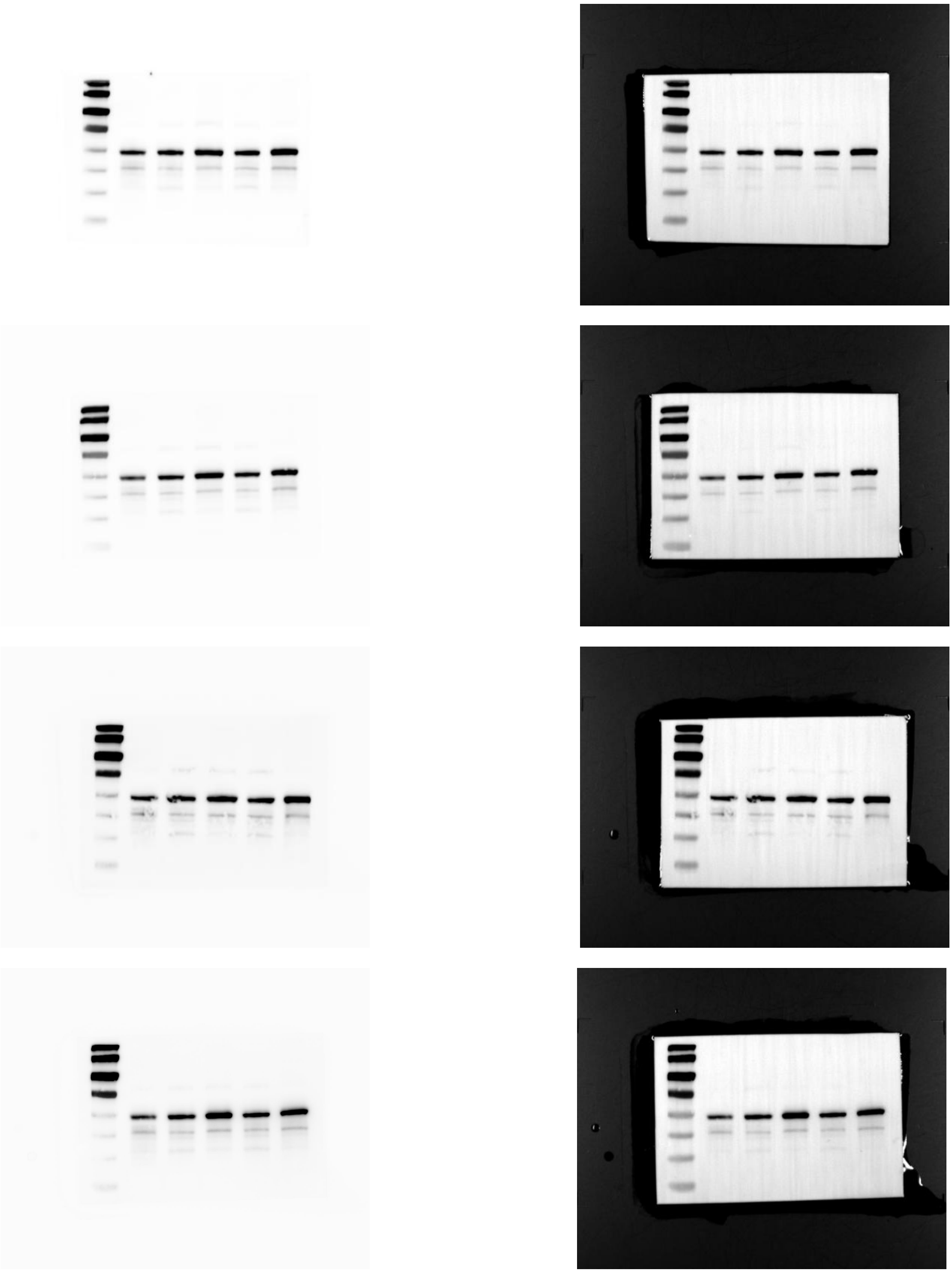

p-PKA

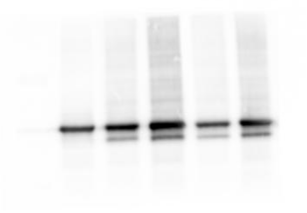
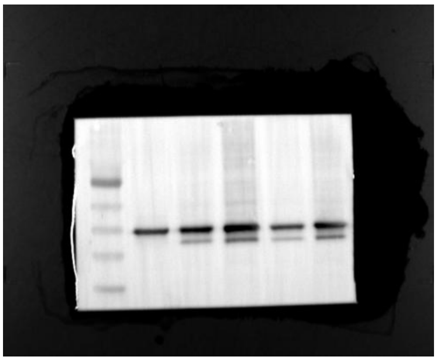
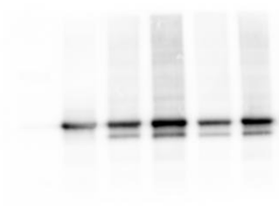
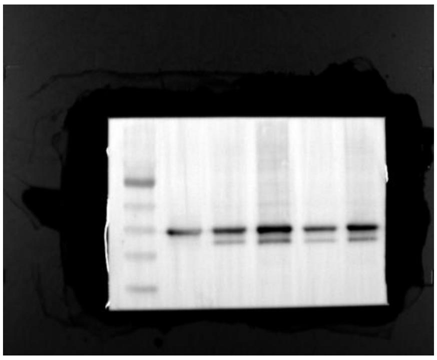
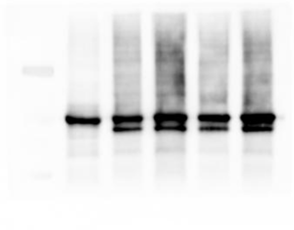
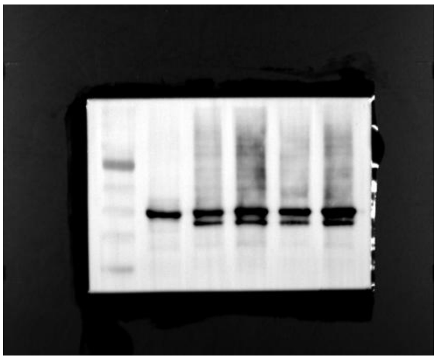
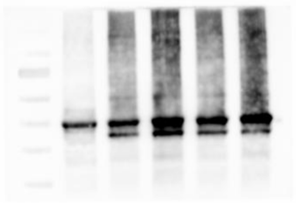
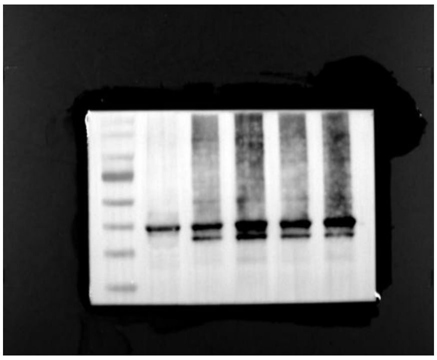
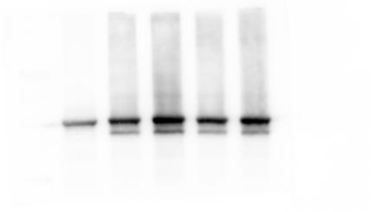
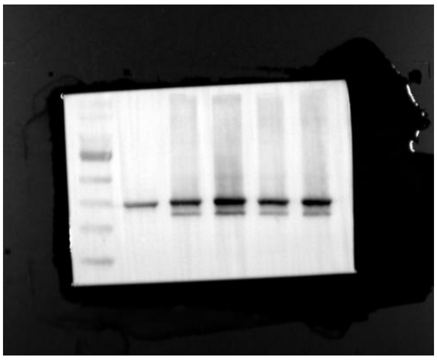

Figure6-A

PKA

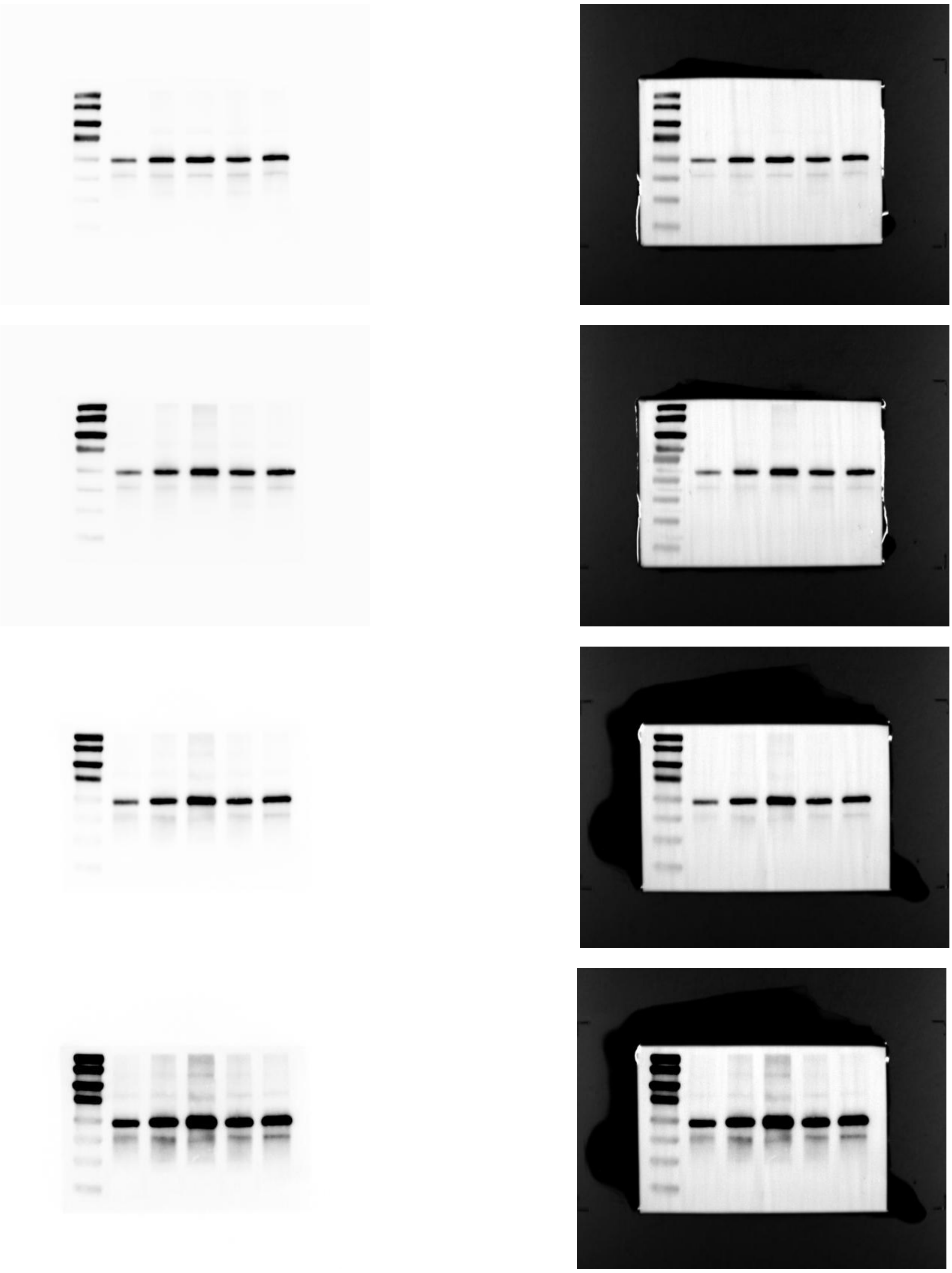

p-PKA

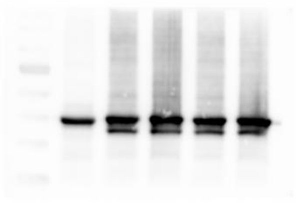

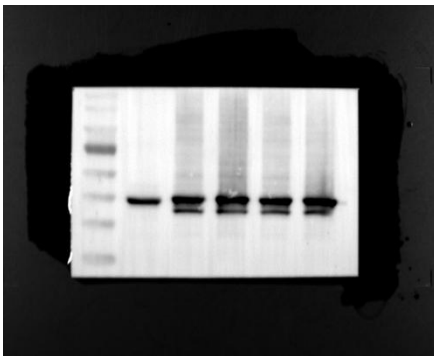

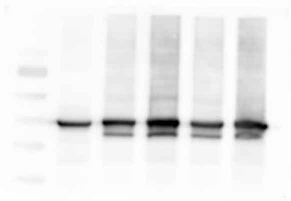

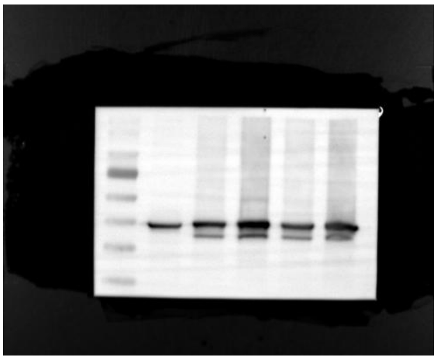

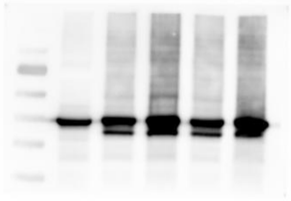

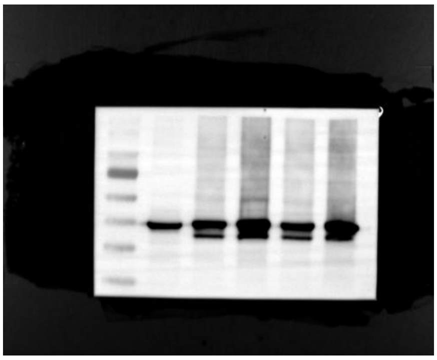

Supplement: Supplementary file 1 — Supplementary Figures. [file 41598_2024_58577_MOESM1_ESM.pdf]
